# Autophagy Orchestrates Anti‐Tumor Immunity to Enhance Chemosensitivity via the FBXW2/C/EBPβ/TIMP‐2 Axis in Colorectal Cancer

**DOI:** 10.1002/advs.77349

**Published:** 2026-08-21

**Authors:** Bing Cheng, Chaoying Wei, Zhitong Niu, Jinxin Chen, Guojie Long, Yingzhen Weng, Qiufeng Liu, Yue Jiang, Panpan Wang, Ronghua Zhang, Qiang Yu, Xiaojian Wu, Wenyu Wang

**Affiliations:** ^1^ Guangdong Provincial Key Laboratory of Colorectal and Pelvic Floor Disease The Sixth Affiliated Hospital Sun Yat‐sen University Guangzhou China; ^2^ Guangdong Institute of Gastroenterology, The Sixth Affiliated Hospital Sun Yat‐sen University Guangzhou China; ^3^ Biomedical Innovation Center, The Sixth Affiliated Hospital Sun Yat‐sen University Guangzhou China; ^4^ Department of General Surgery, The Sixth Affiliated Hospital Sun Yat‐sen University Guangzhou China; ^5^ The First Affiliated Hospital of Jinan University Guangzhou China; ^6^ Pharmacy college Jinan University Guangzhou China; ^7^ Cancer Research Institute Jinan University Guangzhou China; ^8^ Genome Institute of Singapore Agency for Science, Technology, and Research (A*STAR) Biopolis Singapore; ^9^ Department of Physiology, Yong Loo Lin School of Medicine National University of Singapore Singapore Singapore; ^10^ Cancer and Stem Cell Biology Duke‐NUS Medical School Singapore Singapore

**Keywords:** autophagy, cancer research, colorectal cancer, immunity, matrix metalloproteinase, medicine, TIMP‐2

## Abstract

The efficacy of chemotherapy in colorectal cancer (CRC) is closely linked to its ability to remodel the tumor immune microenvironment (TIME), though the underlying molecular determinants remain incompletely defined. Here, we identified tumor‐cell intrinsic autophagy as a pivotal orchestrator of an immunologically active TIME, essential for chemotherapy response. We demonstrated that 5‐FU/Oxaliplatin treatment triggers autophagy‐dependent TIMP‐2 production, which restrains MMP‐2/9 activity and promotes intra‐tumoral accumulation of active CD8^+^ T cells. Disruption of this axis—through autophagy deficiency or loss of TIMP‐2—leads to CD8^+^ T cell exclusion and therapeutic resistance. Conversely, restoration of TIMP‐2 or co‐administration of MMP‐2/9 inhibitor successfully reinvigorated CD8^+^ T cells within the tumor and revived treatment efficacy. Mechanistically, autophagy‐mediated degradation of FBXW2 leads to the accumulation of C/EBPβ via the ubiquitin‐proteasome pathway, thereby driving transcriptional upregulation of TIMP‐2. Clinically, autophagy status and TIMP‐2 expression correlated with favorable immune profiles and improved clinical outcomes. These findings establish the autophagy–TIMP‐2 axis as a key determinant of chemotherapy‐induced immune remodeling and a potential target for enhancing therapeutic response and patient stratification in CRC.

## Graphical abstract

Chemotherapy leverages tumor‐cell autophagy to reshape the colorectal cancer immune microenvironment. Autophagy degrades FBXW2 to promote C/EBPβ‐mediated TIMP‐2 transcription and MMP‐9 suppression, driving intra‐tumoral CD8^+^ T‐cell infiltration. Targeting MMP‐2/9 or restoring TIMP‐2 overcomes chemo‐resistance in autophagy‐deficient tumors.

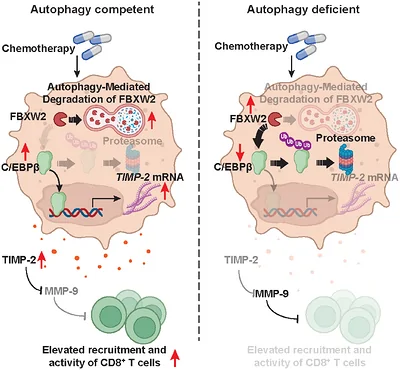

## 1 Introduction

5‐fluorouracil (5‐FU) based regimens such as FOLFOX (5‐FU, leucovorin, and Oxaliplatin) remain the cornerstone for treating patients with colorectal cancer (CRC) [1, 2]. Failure of chemotherapy is a major cause of poor clinical outcome. Beyond its direct cytotoxic activity against tumor cells, accumulating evidence indicated chemotherapy also critically modulated the tumor immune microenvironment (TIME), which in turn substantially affects treatment efficacy. For instance, infiltration and activation of immune cells such as CD8^+^ T cells and macrophages positively correlate with sensitivity to chemotherapy across multiple cancers including CRC [3, 4, 5]. However, the precise mechanisms by which chemotherapy shapes the antitumor immunity in CRC remain poorly understood.

Autophagy is an evolutionarily conserved process playing Janus roles (either tumor‐ suppressing or promoting) during cancer progression in a context‐dependent manner. Loss of key autophagy regulators (e.g., Beclin‐1, ATG5, ATG7) has been associated with tumor initiation in several types of cancers [6, 7, 8]. Conversely, autophagy is exploited by tumor cells to support survival via preserving metabolic fitness and maintaining cellular homeostasis under stress conditions such as hypoxia or therapeutic exposure [9, 10, 11].

Chemotherapy is a known trigger of autophagy, which has been conventionally considered as cytoprotective and a driver of resistance, leading to clinical exploration of autophagy inhibitors such as HCQ, Lys05, and inhibitors against VPS34 or ULK1 [12, 13, 14]. However, these approaches have shown limited success. Paradoxically, accumulating evidence also pinpointed the induction of autophagy enhanced sensitivity of tumor cells to chemotherapeutic agents, including paclitaxel and etoposide. Autophagy inducers such as mTOR inhibitor‐Rapamycin efficiently enhanced chemo‐sensitivity both in vitro and in vivo [15, 16, 17]. Beyond its tumor cell‐autonomous role, autophagy appears to play complex, and at times conflicting, non‐cell‐autonomous roles within the tumor ecosystem. For example, autophagy‐dependent ATP secretion into the tumor microenvironment sent “eating me” signal to innate immune cells such as DC cells, thereby boosting anti‐tumor T cell response and enhancing the efficacy of CD47‐SIRPα blockade [18]. In contrast, autophagy‐mediated degradation of MHC‐I has been defined as an essential mechanism driving immune evasion and ICB resistance in PDAC [19]. These discrepancies highlight the intricate roles of tumor cell–intrinsic autophagy in regulating the tumor ecosystem, including the TIME in response to chemotherapy.

In this study, we identified a tumor cell–intrinsic autophagy pathway that shapes a tumor‐suppressive immune microenvironment during 5‐FU/Oxaliplatin(5‐FU/Oxa) treatment in CRC. Autophagy promoted TIMP‐2 production, which in turn restricted MMP‐2/9 activity, enhanced CD8^+^ T cell infiltration and activation, and ultimately improved therapeutic efficacy. Mechanistically, autophagy‐mediated degradation of the E3 ligase‐FBXW2 stabilized C/EBPβ via the ubiquitin‐proteasome system, therefore driving TIMP‐2 transcription. Inhibition of autophagy or TIMP‐2 abrogated this effect, while restoration of TIMP‐2 signaling or MMP‐2/9 blockade reinvigorated anti‐tumor immunity and enhanced chemosensitivity in mouse models. Clinically, autophagy status correlated with TIMP‐2 expression and patient response to 5‐FU–based chemotherapy. Together, our findings revealed autophagy as a key determinant of immune remodeling and chemosensitivity in CRC.

## 2 Results

### 2.1 Efficacy of Chemotherapy Requires Tumor Cell‐Intrinsic Autophagy in Immunocompetent Hosts

To investigate how autophagy modulates chemotherapy response in colorectal cancer (CRC), particularly from the perspective of antitumor immunity, we used CRISPR/Cas9 to generate CT‐26 cells—a syngeneic microsatellite‐stable (MSS) colon carcinoma line—deficient in the core autophagy regulators ATG5 or ATG7. Ablation of *Atg5/7* effectively blocked 5‐FU/Oxa‐induced autophagy both in vitro and in vivo, as evidenced by: (i) reduced LC3‐I to LC3‐II conversion (Figure S1A‐C), (ii) impaired p62 clearance (Figure S1D), (iii) decreased LC3‐II staining and puncta formation (Figure S1E,F), and (iv) diminished phosphorylation of ATG16L1 at Ser278, a newly described marker that specifically reflects autophagy initiation signals and is predominantly localized to nascent autophagosomes (Figure S1G) [20].

We then implanted these cells into both immunocompetent and immunodeficient mice, followed by treatment with 5‐fluorouracil plus oxaliplatin (5‐FU/Oxa), a combination that mirrors the clinically applied FOLFOX regimen (Figure 1A) [21]. In immunodeficient nude mice lacking functional T cells, *Atg5* knockout (KO) enhanced sensitivity to 5‐FU/Oxa (Figure 1B–D), aligning with the well‐documented cytoprotective role of autophagy in tumor cells under chemotherapy. Strikingly, this effect was reversed in an immunocompetent setting. While wild‐type CT‐26 tumors responded well to 5‐FU/Oxa treatment, *Atg5* KO completely abrogated chemotherapy‐induced tumor shrinkage in immunocompetent mice (Figure 1E–G). Similarly, *Atg7* KO enhanced chemosensitivity in nude mice (Figure 1H–J) but suppressed 5‐FU/Oxa efficacy in immunocompetent hosts (Figure 1K–M). To corroborate these genetic findings pharmacologically, we treated tumor‐bearing immunocompetent mice with the autophagy inhibitor chloroquine (CQ) [19]. While CQ alone had minimal effect on tumor growth, its co‐administration with 5‐FU/Oxa dramatically attenuated therapeutic efficacy, phenocopying the effect of genetic autophagy ablation. Moreover, following the suppression of autophagy by *Atg5* KO in tumor cells, administration of CQ failed to produce an additional reduction in chemosensitivity, suggesting that CQ impairs the chemotherapeutic response directly through its inhibitory effect on tumor cell‐intrinsic autophagy, rather than through indirect regulation of immune cells (Figure 1N–P; Figure S2A). In addition, given that repeated CQ and chemotherapy administration might cause systemic toxicity, we closely monitored mouse body weight and observed that neither monotherapy nor combination therapy resulted in any notable weight alterations (Figure S2B). In parallel, H&E staining of the heart, liver, spleen, lungs, and kidneys from these mice revealed no significant toxic lesions (Figure S2C). Collectively, these data demonstrate that intact autophagy within tumor cells is required for an effective chemotherapy response in immunocompetent CRC models. This suggests autophagy specifically regulates chemotherapy‐induced antitumor immunity—a function that predominates over its canonical cell‐autonomous cytoprotective role.

**FIGURE 1:**
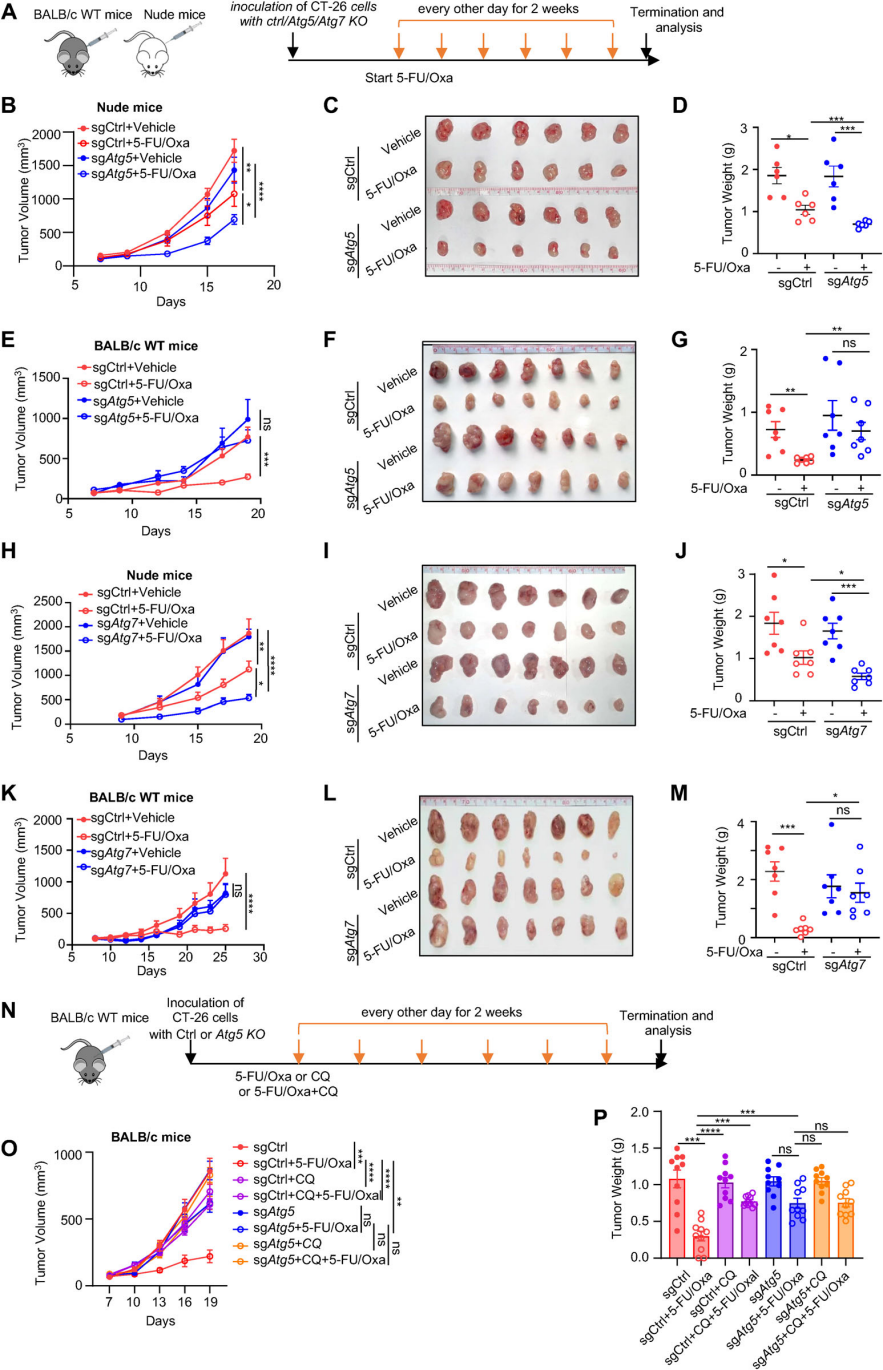
Tumor cell‐intrinsic autophagy is required for efficacy of chemotherapy in immunocompetent host. (A) Schematic representation of the treatment schedule in BALB/c nude and wild‐type (WT) mice bearing subcutaneous CT‐26 tumors with or without *Atg5/Atg7* knockout (sgCtrl vs sg*Atg5*/sg*Atg7*). All drugs (5‐FU, 25 mg/kg; Oxa, 2.5 mg/kg) were administered intraperitoneally (i.p.) every other day for 2 weeks. (B–G) Growth curves of tumors, representative images, and tumor weights for CT‐26 tumors with or without sg*Atg5* in nude (B–D) or WT (E–G) mice (n = 6–7 per group). (H–M) Growth curves of tumors, representative images, and tumor weights for sgCtrl versus sg*Atg7* tumors in nude (H–J) or WT (K–M) mice (n = 7 per group). (N) Treatment scheme for CT‐26 tumors with or without sg*Atg5* in WT mice for assessing the efficacy of 5‐FU/Oxa, CQ alone, or in combination (n = 8‐10 per group). All drugs were administered intraperitoneally (i.p.) every other day for 2 weeks; CQ was given at a dose of 60 mg/kg. (O) Tumor growth curves, and (P) tumor weights for the indicated treatment groups. Data are shown as mean ± SEM. Statistical analysis was performed using Two‐way ANOVA with multiple comparisons. Significance is denoted as ^*^ *p* < 0.05, ^**^ *p* < 0.01, ^***^ *p* < 0.001, ^****^ *p* < 0.0001, ns, not significant.

### 2.2 Tumor Cell‐Intrinsic Autophagy Shapes a Tumor‐Suppressive Immune Microenvironment Upon 5‐FU/Oxa Treatment

To dissect the role of autophagy in modulating the immune response elicited by 5‐FU/Oxa, we profiled the tumor immune microenvironment (TIME) using mass cytometry by time‐of‐flight (CyTOF) with 37‐immune lineage markers in tumors with/without *Atg5* KO upon 5‐FU/Oxa treatment or not. Using t‐distributed stochastic neighbor embedding (t‐SNE) analysis, we identified 36 distinct cell clusters and reclassified them to 10 major immune cell subgroups, including T cells, NK cells, B cells, macrophages, and other myeloid cells [22, 23] (Figure S3A–D). 5‐FU/Oxa treatment significantly increased the proportions of CD8^+^ T cells and NK cells along with a substantial reduction in macrophages, indicating an active antitumor immune response. In contrast, autophagy inhibition via *Atg5* KO specifically attenuated the 5‐FU/Oxa‐induced expansion of CD8^+^ T cells, while no significant alterations in NK cells or macrophages were observed (Figure 2A; Figure S3E,F). Similarly, flow‐cytometry and immunohistochemistry (IHC) analysis further confirmed that abrogation of autophagy by *Atg5/7* KO remarkably reduced CD8^+^ T cell infiltration in tumors induced by 5‐FU/Oxa (Figure 2B,C). To gain a deeper insight into the function of T cells, CD8^+^ T and CD4^+^ T cells were re‐classified according to their respective functions using established markers [24, 25, 26]. The analysis revealed 7 distinct subtypes of CD8^+^ T and CD4^+^ T cells: Naïve CD8^+^ T (C05; CD8^+^ CD44^Low^), Ly6C^−^ CD8^+^ Tem (C14; CD8^+^ Ly6C^−^ CD44^High^ CD62L^−^), Ly6C^+^ CD8^+^ Tem (C08, C11‐13; CD8^+^ Ly6C^+^ CD44^High^ CD62L^−^), Ly6C^+^ CD8^+^ Tcm (C10; CD8^+^ Ly6C^+^ CD44^High^ CD62L^+^), Naïve CD4^+^ T (C06; CD4^+^ CD44^Low^), Treg (C07, C18; CD4^+^ CD44^High^ CD25^High^ Foxp3^High^), and ICOS^+^ PD1^+^ CD4^+^ T (C09, C17; CD4^+^ CD44^High^ CD25^Low^ Foxp3^Low^ ICOS^+^ PD1^+^) (Figure S4). Notably, the proportion of Ly6C^+^CD8^+^ Tem cells increased following 5‐FU/Oxa treatment, an effect that was markedly suppressed upon autophagy inhibition (Figure 2D). The 5‐FU/Oxa‐induced elevation of Ly6C^+^ CD8^+^ T cells was further verified by flow‐cytometry, whereas this trend was substantially curtailed upon autophagy inhibition by either *Atg5* or *Atg7* KO (Figure 2E). Previous studies have illustrated that Ly6C^+^ CD8^+^ T cells execute effector/memory T cell functions and display cytotoxic activity with elevated expression of activation markers including Granzyme B(GZMB) [27, 28]. In line with this, flow cytometry and IHC analyses confirmed that 5‐FU/Oxa treatment markedly increased the frequencies of GZMB^+^, perforin^+^, and TNFα^+^ CD8^+^ T cells, an effect that was largely abrogated in *Atg5‐* or *Atg7*‐deficient tumors (Figure 2F; Figure S5). Of note, these Ly6C^+^ CD8^+^ T cells also displayed enhanced expression of activation markers (CD69, GZMB, CD103, CD86) and reduced expression of inhibitory markers (PD‐1, TIM‐3) (Figure 2G) [29, 30, 31, 32]. To further investigate the direct impact of tumor cell‐intrinsic autophagy on CD8^+^ T cells, we conducted in vitro co‐culture experiments. Splenic CD8^+^ T cells from OT‐1 mice were pre‐activated with OVA peptide and then co‐cultured with tumor interstitial fluid (TIF) derived from CT‐26 sg*Ctrl* or sg*Atg5* tumors, with or without 5‐FU/Oxa treatment. TIF from 5‐FU/Oxa‐treated CT‐26 sg*Ctrl* tumors significantly promoted the proliferation and activation of CD8^+^ T cells, as assessed by CFSE dilution and the key functional markers including CD69, TNF‐α, and IFN‐γ. Conversely, this effect was markedly attenuated when TIF was derived from sg*Atg5* tumors (Figure 2H–L; Figure S6). Consistently, CD8^+^ T cells pre‐treated with TIF from 5‐FU/Oxa‐treated CT‐26 sg*Ctrl* tumors exhibited enhanced cytotoxic activity against tumor cells compared with the control group, while *Atg5* KO substantially impaired their killing efficacy (Figure 2M). Taken together, these results suggested that 5‐FU/Oxa provoked anti‐tumor immune response by driving CD8^+^ T cell proliferation and activation, which depended on tumor cell‐intrinsic autophagy.

**FIGURE 2:**
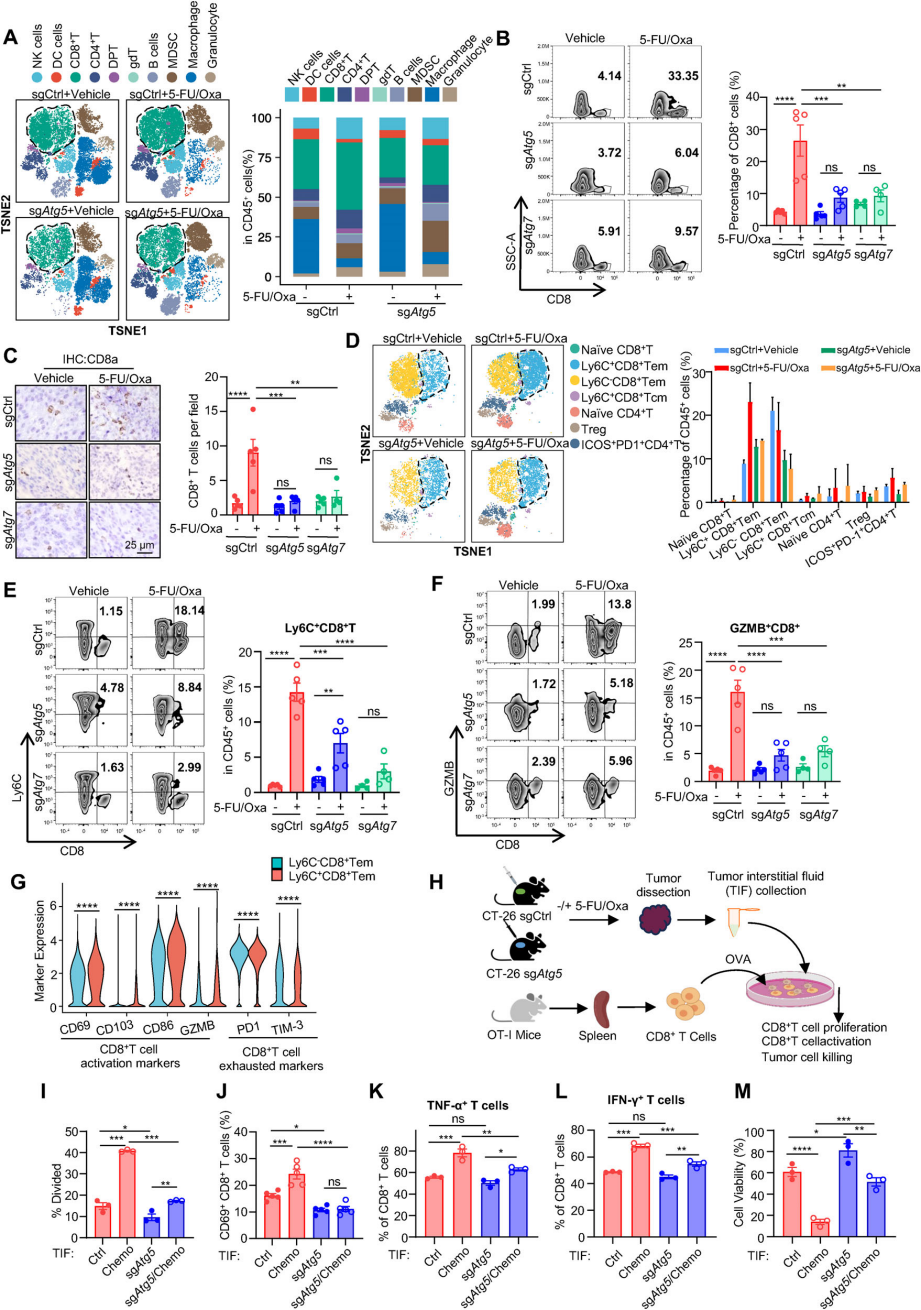
Anti‐tumor immunity evoked by chemotherapy depends on tumor cell‐intrinsic autophagy. (A) t‐SNE plots (left) and the frequency (right) of immune cell subsets in each treatment group, as determined by CyTOF (n = 2 per group). Each dot represents a single cell, with cell types color‐coded. (B) Flow cytometric analysis and (C) immunohistochemical (IHC) staining of CD8^+^ T cells in sg*Atg5* or sg*Atg7* tumors following 5‐FU/Oxa treatment (scale bar: 25 µm). (D) t‐SNE plots (left) and quantification (right) of CD8^+^ and CD4^+^ T‐cell subpopulations identified by CyTOF. (E,F) Flow cytometric analysis of activated CD8^+^ T cells (Ly6C^+^ or GZMB^+^) and quantitative summary of the indicated subsets. (G) Relative expression levels of activation and exhaustion markers in Ly6C^+^ versus Ly6C^−^ CD8^+^ T cells, assessed by CyTOF. (H) Schematic showing of the in vitro co‐culture system using spleen‐derived CD8^+^ T cells and tumor interstitial fluid (TIF) from sgCtrl or sg*Atg5* tumors. (I) CFSE staining analysis of CD8^+^ T cells proliferation for the indicated treatment groups. (J–L) Flow cytometric analysis of CD69^+^CD8^+^ T, TNF‐α^+^CD8^+^ T, and IFN‐γ^+^CD8^+^ T cells from the indicated groups. (M) The relative cell viability of tumor cells after co‐culturing with CD8^+^ T cells treated the indicated TIF were measured by cell‐titer assay. Data are shown as mean±SEM. Statistical analysis was performed using Unpaired t‐test (in G) and Two‐way ANOVA with multiple comparisons for others. Significance is indicated as ^*^ *p* < 0.05, ^**^ *p* < 0.01, ^***^ *p* < 0.001, ^****^ *p* < 0.0001, ns, not significant.

### 2.3 TIMP‐2 Serves as a Crucial Downstream Effector of Autophagy‐Mediated Chemo‐Sensitivity

To explore the underlying mechanisms linking 5‐FU/Oxa induced autophagy and anti‐tumor immunity, we first performed RNA‐seq using RKO cells with or without autophagy inhibition upon exposure to 5‐FU/Oxa or not. Totally, 1683 genes were upregulated upon treatment with 5‐FU/Oxa, and 300 genes were downregulated after autophagy inhibition by knocking down *ATG5*. Intersection of these two gene sets yielded 172 genes that were upregulated by 5‐FU/Oxa in an autophagy‐dependent manner. GO enrichment analysis revealed that pathways related to cytokine production were highly enriched among these genes (Figure S7). Given that cytokines serve as key mediators that are secreted into the tumor microenvironment (TME) to modulate antitumor immunity, we further performed a membrane‐based cytokine array using culture supernatants from RKO cells treated with 5‐FU/Oxa, with or without autophagy inhibition. A total of seven cytokines—PLGF, LIF, MIF, TIMP‐2, IL‐8, GRO, and IGFBP‐2—were identified as potential candidates upregulated by 5‐FU/Oxa treatment in an autophagy‐dependent manner (Figure 3A). Among these, LIF, MIF, IL‐8, GRO, and IGFBP‐2 have been previously reported to be regulated by autophagy in a cell‐context dependent manner, whereas the relationship between PLGF or TIMP‐2 and autophagy remains unclear [33, 34, 35]. TIMP‐2, a secreted endogenous inhibitor of MMP‐2/9, attracted our particular interest because its low expression has been associated with poor prognosis in colorectal cancer clinical samples [36]. However, its role in modulating the tumor immune microenvironment (TIME) and influencing chemosensitivity remains largely unknown. ELISA assay confirmed that TIMP‐2 secretion induced by 5‐FU/Oxa required intact autophagy (Figure 3B). To determine the role of TIMP‐2 upon 5‐FU/Oxa treatment, we established CT‐26 cells with *Timp‐2* knockout, and the efficacy was validated by WB analysis. Interestingly, *Timp‐2* KO in CT‐26 cells markedly impaired response to 5‐FU/Oxa in immunocompetent mice, phenocopying the loss of autophagy (Figure 3C–E). In contrast, *Timp‐2* KO had no significant effect on the response to 5‐FU/Oxa in immunodeficient nude mice, indicating TIMP‐2 as a pivotal regulator of anti‐tumor immunity to modulate response to 5‐FU/Oxa (Figure 3F–H). Conversely, ectopic expression of TIMP‐2 in *Atg5* KO CT‐26 cells can efficiently restore chemosensitivity *in* immunocompetent mice (Figure 3I–L). Together, these findings defined TIMP‐2 as a critical downstream effector through which autophagy enhances CRC sensitivity to 5‐FU/Oxa.

**FIGURE 3:**
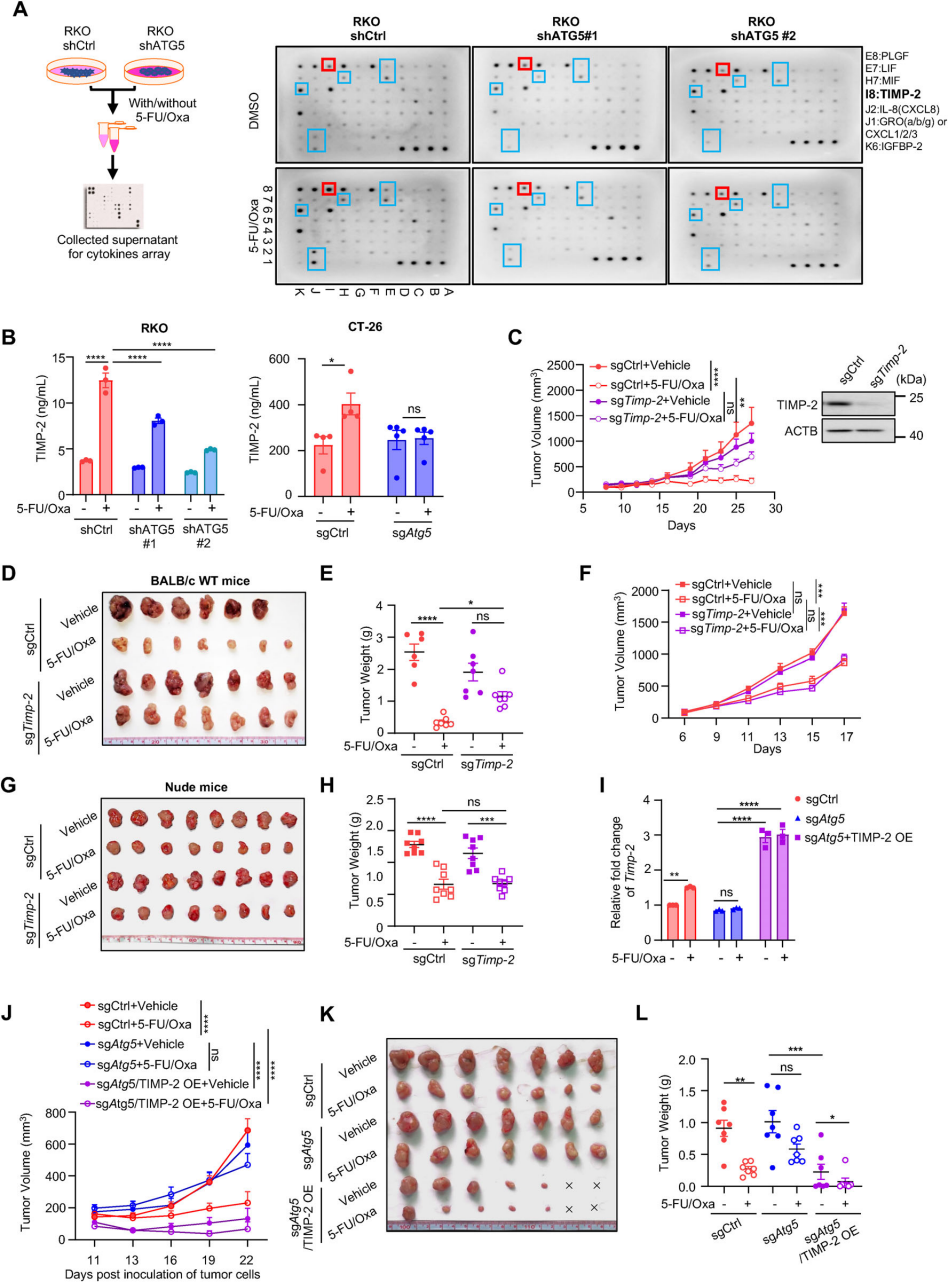
TIMP‐2 is a downstream effector of the autophagy‐mediated response to chemotherapy. (A) Left: Schematic workflow for collecting cell culture supernatant from RKO cells expressing shCtrl or shATG5 after 48 h treatment with 5‐FU/Oxa (100 µM/2.5 µM). Right: Membrane‐based cytokine antibody array of secreted cytokines from the indicated groups; boxed spots indicate cytokines induced by 5‐FU/Oxa in an autophagy‐dependent manner. (B) ELISA quantification of TIMP‐2 levels in culture supernatants from RKO and CT‐26 cells with or without ATG5 silencing under 5‐FU/Oxa treatment. (C–H) Growth curves of tumors, representative images, and tumor weights for CT‐26 tumors with or without *Timp‐2* knockout(sgCtrl vs sg*Timp‐2)* in WT (C–E) or nude (F–H) mice (n = 6‐8 per group). Timp‐2 knockout was confirmed by WB. (I) Generation of autophagy‐deficient CT‐26‐sg*Atg5* cells with or without ectopic TIMP‐2 expression; TIMP‐2 mRNA was confirmed by qRT‐PCR. (J–L) Growth curves of tumors, representative images, and tumor weights for CT‐26 tumors from sgCtrl or sg*Atg5* cells with or without TIMP‐2 overexpression (TIMP‐2 OE), under the indicated treatments. Data are shown as mean±SEM. Statistical analysis was performed using Two‐way ANOVA with multiple comparisons. Significance is indicated as ^*^ *p* < 0.05, ^**^ *p* < 0.01, ^***^ *p* < 0.001, ^****^ *p* < 0.0001, ns, not significant.

### 2.4 TIMP‐2 Mediates Chemosensitivity by Promoting CD8^+^ T Cell Activation

To further elucidate how TIMP‐2 modulates the immune response, we first examined the proportion of tumor‐infiltrating CD8^+^ T cells by IHC staining. Similar to autophagy inhibition, *Timp‐2* KO also led to a substantial reduction in CD8^+^ T abundance (Figure 4A). In addition, the 5‐FU/Oxa‐induced increases in Ly6C^+^ CD8^+^ T and GZMB^+^ CD8^+^ T cells were dramatically impaired upon *Timp‐2* KO (Figure 4B,C). Conversely, overexpression of TIMP‐2 in autophagy‐deficient CRC cells can efficiently rescue the abundance of total CD8^+^ T and Ly6C^+^ CD8^+^ T cell populations in the tumor sections (Figure 4D,E). Accordingly, the proportions of GZMB^+^ CD8^+^ T cells also exhibited a remarkable increase after TIMP‐2 reconstitution, as assessed by flow‐cytometry and immunofluorescence (IF) analysis, which correlated with the reduced tumor burden (Figure 4F; Figure S8). Importantly, the chemosensitivity restored by TIMP‐2 overexpression was substantially abrogated upon CD8^+^ T cell depletion using neutralizing antibodies, confirming that TIMP‐2 regulates chemosensitivity in a CD8^+^ T cell‐dependent manner (Figure 4G–J). To delineate the role of TIMP‐2 in regulating CD8^+^ T cells, we performed in vitro co‐culture experiments. Compared with TIF from treatment‐naïve tumors, TIF from chemo‐treated tumors significantly enhanced CD8^+^ T cell activation, whereas this effect was dramatically weakened after *Timp‐2* KO (Figure 4K). Furthermore, TIMP‐2 antibody blockade markedly attenuated CD8^+^ T cell activation induced by TIF from chemo‐treated tumors (Figure 4L). Conversely, the addition of recombinant TIMP‐2 successfully reinvigorated CD8^+^ T cell activity that was suppressed upon stimulation with TIF from CT‐26 sg*Atg5* tumors. However, treatment with TIMP‐2 alone did not further enhance CD8^+^ T cell activation, suggesting that TIMP‐2 indirectly acts on the TIF to reactivate anti‐tumor immunity (Figure 4M). Collectively, these results identified TIMP‐2 as a key mediator of 5‐FU/Oxa sensitivity by promoting functional CD8^+^ T cells.

**FIGURE 4:**
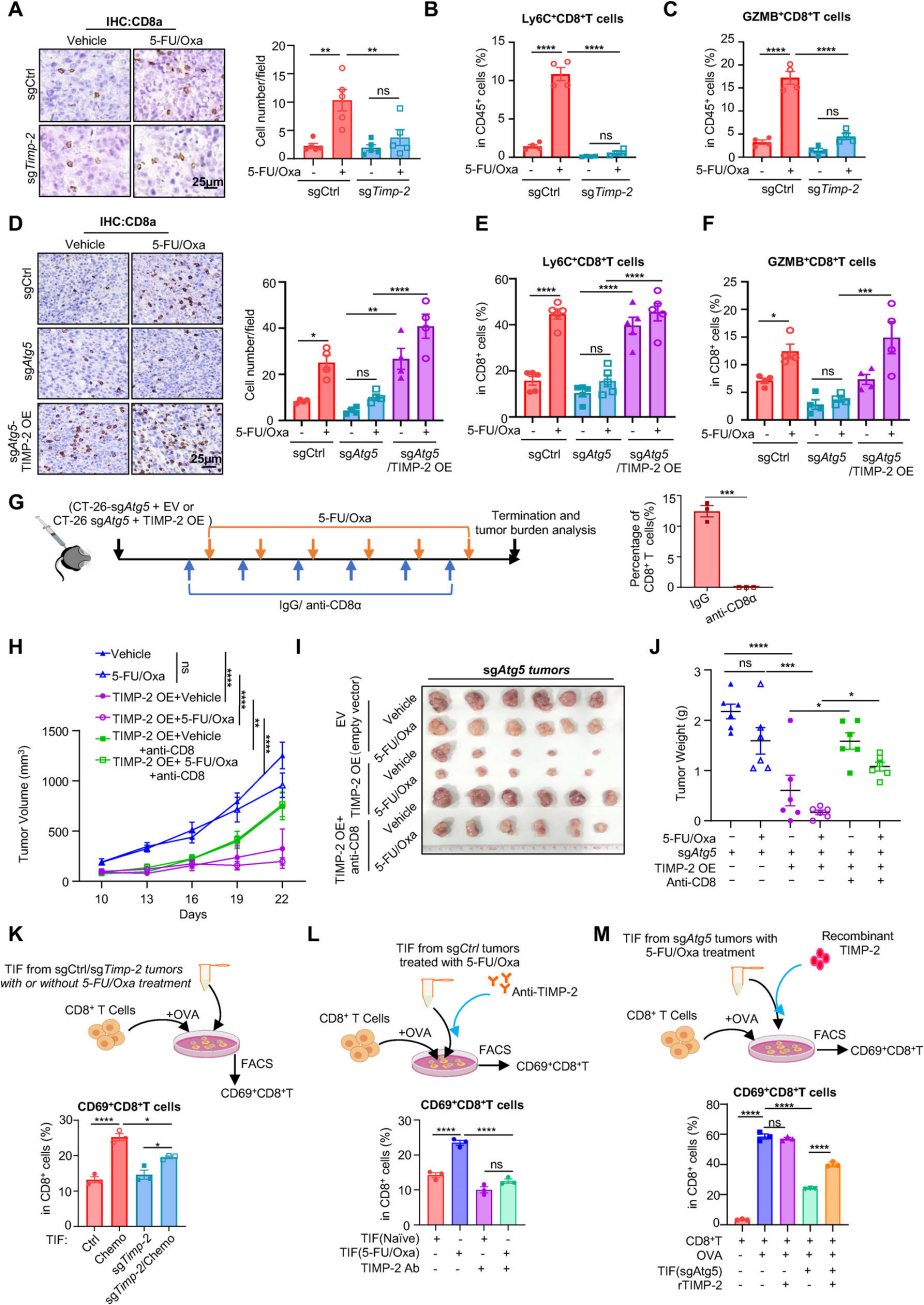
TIMP‐2 rescues chemotherapy sensitivity through activation of CD8^+^ T cells. (A) Representative IHC images(left) and quantification(right) of infiltrated CD8^+^ T cells in control or TIMP‐2 knockout tumors treated with vehicle or 5‐FU/Oxa (scale bar: 25 µm). (B,C) Flow cytometric quantification of activated CD8^+^ T cell subsets (Ly6C^+^ or GZMB^+^) in the same groups as (A). (D) Representative IHC images and quantification of CD8^+^ T cell infiltration in CT‐26 sgCtrl, sg*Atg5*, and sg*Atg5*/TIMP‐2 OE tumors under vehicle or 5‐FU/Oxa treatment (scale bar: 25 µm). (E,F) Flow cytometric quantification of activated CD8^+^ T populations (Ly6C^+^ and GZMB^+^) for the groups in (D). (G) Schematic of the treatment protocol for CD8^+^ T‐cell depletion in BALB/c WT mice, with depletion efficiency verified by FACS. (H–J) Tumor growth curves, representative tumor images, and tumor weights at endpoint for mice bearing CT‐26‐sg*Atg*5 tumors with or without TIMP‐2 OE, subjected to the indicated treatments (with or without CD8 depletion) (n = 5‐6 per group). (K) CD8^+^ T cells were pre‐incubated with tumor interstitial fluid (TIF) from the indicated tumors and then stimulated with OVA peptide; activation was assessed by FACS for CD69^+^ CD8^+^ T cells. (L) CD8^+^ T cells were incubated with TIF from treatment‐naïve or chemo‐treated tumors, with or without TIMP‐2 neutralizing antibody, and CD69^+^ CD8^+^ T cells were quantified by FACS. (M) CD8^+^ T cells were incubated with TIF from chemo‐treated sg*Atg5* tumors supplemented with recombinant TIMP‐2 or vehicle, and CD69^+^CD8^+^ T cells were analyzed by FACS. Data are shown as mean ± SEM. Statistical analysis was performed using Unpaired t‐test (in G), One‐way ANOVA with multiple comparisons (in M), and Two‐way ANOVA with multiple comparisons for others. Significance is indicated as ^*^ *p* < 0.05, ^**^ *p* < 0.01, ^***^ *p* < 0.001, ^****^ *p* < 0.0001, ns, not significant.

### 2.5 TIMP‐2 Promotes Anti‐Tumor Immunity and Chemo‐Sensitivity via Modulating MMP‐2/9 Activity

Next, we sought to investigate how TIMP‐2 contributes to anti‐tumor immune response induced by 5‐FU/Oxa. TIMP‐2 is an endogenous matrix metallopeptidase (MMP) inhibitor, which preferentially inhibits the activity of MMP‐2/9 [37, 38]. This prompted us to test whether TIMP‐2 could provoke anti‐tumor immunity and enhance efficacy of 5‐FU/Oxa through MMP‐2/9. To this end, we generated autophagy‐deficient CT‐26 (sg*Atg5*) cells expressing either wild‐type TIMP‐2 or loss‐of‐function mutants (TIMP‐2 Ala+ mutant and TIMP‐2 N‐terminal truncation mutant, hereafter referred to as TIMP‐2 Ala^+^ MUT and TIMP‐2‐C, respectively), which lack MMP inhibitory activity [39, 40, 41]. Of note, Quantitative real‐time PCR (qRT‐PCR), WB, and ELISA analysis of TIMP‐2 revealed that wild‐type TIMP‐2 (TIMP‐2 WT) and the two loss‐of‐function mutants exhibited comparable expression levels (Figure 5A,B). Intriguingly, Only TIMP‐2 WT restored sensitivity to 5‐FU/Oxa in *Atg5‐*KO CT‐26 tumors, whereas both TIMP‐2 mutants failed to do so, suggesting that TIMP‐2 mediated sensitivity to 5‐FU/Oxa relies on its ability to modulate MMP‐2/9 activity (Figure 5C–E). Consistently, only TIMP‐2 WT, but not the two mutants, rescued abundance of total and activated CD8^+^ T cells that was substantially suppressed by autophagy loss (Figure 5F,G). Furthermore, an in vitro assay revealed that MMP‐9 impaired activation of CD8^+^ T cells induced by OVA, while addition of recombinant TIMP‐2 reversed this effect, indicating a role of TIMP‐2 to activate CD8^+^ T cells through inhibiting MMP‐9 activity (Figure 5H). More importantly, combined administration of the small‐molecule inhibitor SB‐3CT, which targets MMP‐2/9 [42], significantly enhanced chemosensitivity in autophagy‐deficient mouse models, whereas such an effect was not evident in the control group (Figure 5I–K). This discrepancy may be due to the fact that, in the wild‐type group, chemotherapy had already induced TIMP‐2, which suppressed MMP‐2/9 activity; therefore, the addition of the inhibitor did not produce a significant sensitizing effect. Consistently, the proportions of total and active CD8^+^ T cells were largely restored by SB‐3CT, particularly in autophagy‐deficient mouse models, positioning MMP‐2/9 as essential modulators of sensitivity to 5‐FU/Oxa via regulating immune response (Figure 5L–N). Furthermore, we monitored body weight changes and performed H&E staining of the heart, liver, spleen, lung, and kidney to assess drug‐related toxicity, and found that neither single‐agent nor combination treatment caused significant systemic toxicity (Figure S9A,B). Interestingly, aligning with our findings, a recent study demonstrated that in hepatocellular carcinoma (HCC) MMP‐9 can suppress infiltration and cytotoxicity of CD8^+^ T cells, therefore improving the efficacy of anti‐PD‐1 [31]. Taken together, our results demonstrated that TIMP‐2 mediated sensitivity to 5‐FU/Oxa and anti‐tumor immune response through modulating MMP‐2/9 activity, and highlighted MMP‐2/9 blockade as a promising strategy to restore chemosensitivity in CRC with dysfunctional autophagy.

**FIGURE 5:**
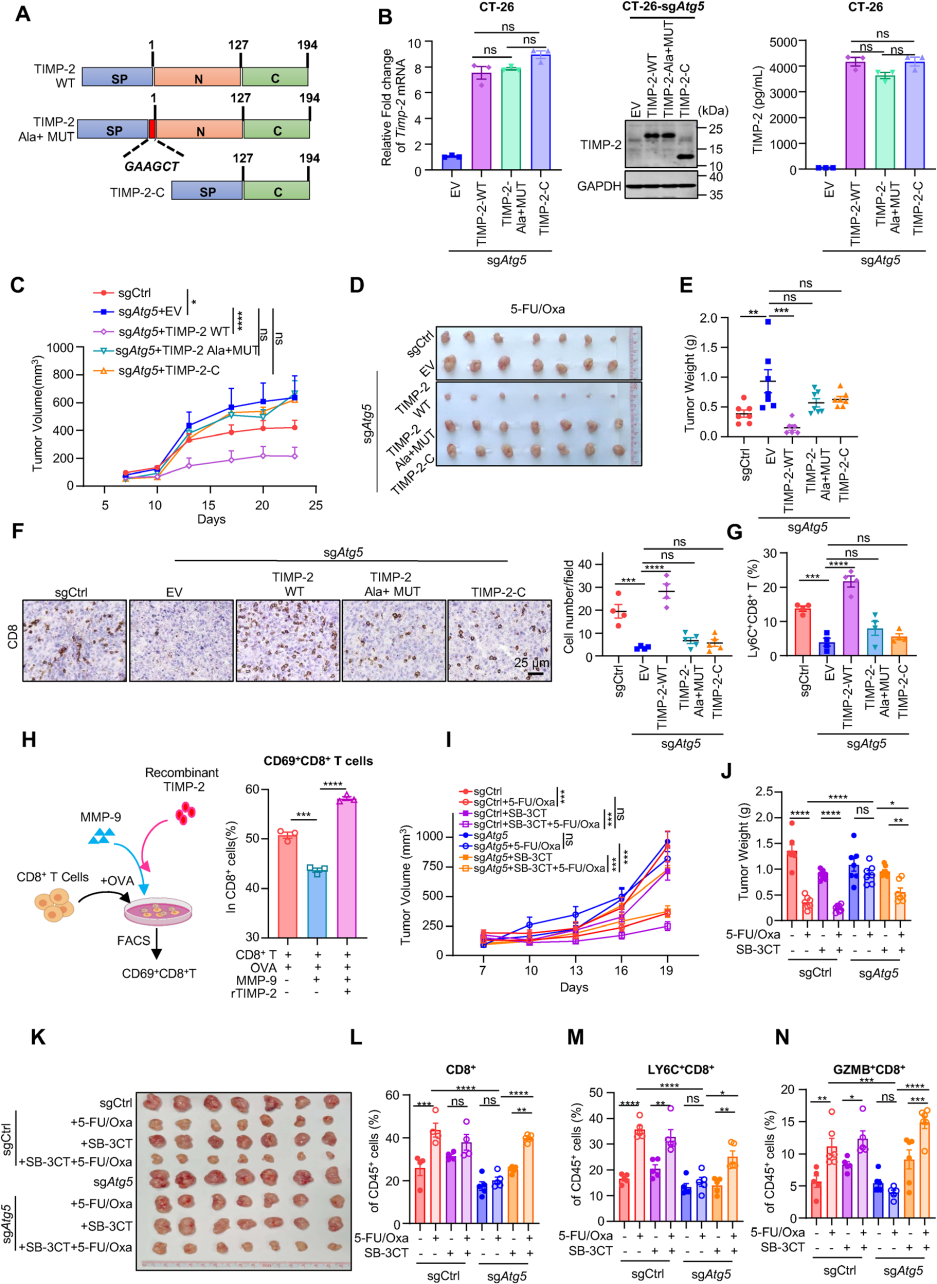
Autophagy‐TIMP‐2 axis regulates anti‐tumor immunity triggered by chemotherapy via modulating MMP‐2/9 activity. (A) Schematic diagram showing TIMP‐2 wild‐type (WT) and its loss‐of‐function mutants, which are TIMP‐2 mutant with an extra alanine—TIMP‐2 Ala^+^ MUT and N terminal truncated mutant‐TIMP‐2‐C. (B) The expression of TIMP‐2 was confirmed by qRT‐PCR, WB and ELISA. C) CT‐26‐sg*Ctrl* or sg*Atg5* cells harboring TIMP‐2 WT, TIMP‐2 Ala^+^ MUT, TIMP‐2‐C or empty vector (EV) as control were inoculated into BALB/c WT mice and subjected to the 5‐FU/Oxa treatment. Tumor growth curves were monitored over time. (D,E) Representative tumor images and quantitative analysis of tumor weights at sacrifice for the indicated groups (n = 7 per group). (F) Representative IHC images and quantification of intratumoral CD8^+^ T cell infiltration in the indicated groups as in (C) following 5‐FU/Oxa treatment. Scale bar:25 µm. (G) FACS analysis of the proportions of active Ly6C^+^CD8^+^ T cells in the indicated groups, as in (F). (H) CD8^+^ T cells were incubated with active MMP‐9 or in combination with recombinant TIMP‐2, followed by OVA stimulation. Activation of CD8^+^ T cells was assessed by FACS. (I) CT‐26‐sg*Ctrl* or sg*Atg5* cells were inoculated into BALB/c WT mice and treated with 5‐FU/Oxa, MMP‐2/9 inhibitor SB‐3CT (10 mg/kg, i.p.) alone or in combination. Tumor growth curves were established. (J,K) Quantification of tumor weights at sacrifice and representative tumor images for the indicated groups in(I) (n = 7 per group). (L–N) FACS analysis of total and active CD8^+^ T cells within tumors from the indicated groups in (I). Data are shown as mean ± SEM. Statistical analysis was performed using One‐way ANOVA with multiple comparisons. Significance is indicated as ^*^ *p* < 0.05, ^**^ *p* < 0.01, ^***^ *p* < 0.001, ^****^ *p* < 0.0001, ns, not significant.

### 2.6 The FBXW2‐C/EBPβ Axis Mediates Autophagy‐Dependent Upregulation of TIMP‐2 in Response to 5‐FU/Oxa Treatment

To clarify how autophagy regulates TIMP‐2 expression upon 5‐FU/Oxa treatment, we first assessed TIMP‐2 mRNA levels. Consistent with our RNA‐seq data, which implicated autophagy in cytokine upregulation, autophagy inhibition markedly attenuated the 5‐FU/Oxa‐induced increase in TIMP‐2 mRNA, suggesting that autophagy regulates TIMP‐2 expression at the transcriptional level (Figure 6A). Given that autophagy is a conserved process responsible for cargo degradation, we performed TMT‐labeled proteomic profiling on CRC cells treated with 5‐FU/Oxa, with or without autophagy inhibition. This analysis identified 467 autophagy‐dependent differentially expressed proteins (DEPs), including 246 up‐regulated and 221 down‐regulated proteins (Figure 6B). To screen for candidate transcription factors governing TIMP‐2 expression, we intersected the set of 246 up‐regulated proteins with three publicly available datasets containing predicted TIMP‐2 transcription factors [43]. As a result, CCAAT/enhancer‐binding protein beta (C/EBPβ, encoded by the *CEBPB* gene) emerged as the unique candidate (Figure 6C). Consistent with our findings, clinical data revealed a strong positive correlation between CEBPB and TIMP‐2 expression in CRC patients (Figure 6D). To determine whether the increase in TIMP‐2 expression was attributable to C/EBPβ accumulation following 5‐FU/Oxa treatment, we transfected RKO, SW620, and CT‐26 cells with two independent siRNAs targeting CEBPB and measured TIMP‐2 mRNA levels by qRT‐PCR. As expected, knockdown of C/EBPβ profoundly suppressed the 5‐FU/Oxa‐induced expression of TIMP‐2 (Figure 6E). Conversely, ectopic expression of C/EBPβ significantly promoted the transcription of TIMP‐2 (Figure 6F). These data strongly indicated that C/EBP‐β could serve as a critical transcription factor required for the 5‐FU/Oxa‐elicited upregulation of TIMP‐2. Moreover, in silico analysis of the TIMP‐2 promoter region using JASPAR revealed two consensus C/EBPβ binding sites located at −1755 to −1764 (BS1) and −501 to −511 (BS2), both with scores >8.5, suggesting that TIMP‐2 is a direct transcriptional target of C/EBPβ. To validate this, we constructed plasmids containing the full‐length *TIMP‐2* promoter region or two mutants with depletion of either C/EBPβ binding site (hereinafter referred to as *TIMP‐2* FL, *TIMP‐2* BS1 Del, and *TIMP‐2* BS2 Del) and performed a dual‐luciferase‐based reporter assay. Interestingly, *TIMP‐2* FL was significantly responsive to 5‐FU/Oxa treatment, whereas deletion of either BS1 or BS2 markedly attenuated this response, indicating that both binding sites are essential for the regulation of TIMP‐2 stimulated by 5‐FU/Oxa (Figure 6G). Consistently, chromatin immunoprecipitation (ChIP) assay confirmed that C/EBPβ strongly bound both binding sites, particularly upon treatment with 5‐FU/Oxa (Figure 6H). Taken together, these findings established C/EBPβ as an essential transcription factor for TIMP‐2 upregulation. To dissect the mechanistic link between autophagy and 5‐FU/Oxa‐induced C/EBP‐β accumulation, we next interrogated E3 ligases represented within the 221 down‐regulated proteins (Figure 6I). Of note, among the eight downregulated E3 ligases, the top hit—TRAF6—has been reported to be degraded via autophagy in a p62‐dependent manner, supporting the reliability of our analysis [16, 30, 44]. Since LIR (LC3‐interacting Region) motif is important for autophagy mediated cargo degradation [45], we performed an in silico prediction using the iLIR database (https://ilir.warwick.ac.uk). FBXW2 was identified as the E3 ligases with the highest number of LIR motifs, suggesting that it could be a direct substrate of autophagy (Figure S10A). Intriguingly, further sequence analysis revealed that C/EBPβ possessed an evolutionarily conserved amino‐acid sequence (SLS *TS* SSS *S* **S**P) that resembled the canonical FBXW2 degradation motif (*TS* XXX *S*), containing residues for a prerequisite phosphorylation of substrates [46, 47, 48, 49, 50] (Figure 6J). These findings strongly suggest that autophagy‐mediated degradation of FBXW2 leads to the accumulation of its potential substrate C/EBPβ, thereby accounting for the 5‐FU/oxaliplatin‐induced upregulation of TIMP‐2. To validate our mass spectrometry data, we performed WB analysis in RKO, SW620 and CT‐26 cells. The results confirmed that 5‐FU/Oxa treatment promoted FBXW2 degradation, accompanied by C/EBPβ accumulation, whereas ATG5 KO substantially suppressed this effect (Figure 6K). Additionally, the lysosomal inhibitor bafilomycin A1(Baf A1) effectively reversed both the reduction of FBXW2 and C/EBPβ accumulation induced by 5‐FU/Oxa (Figure 6L). Furthermore, co‐localization of FBXW2 and the LC3 puncta markedly increased upon 5‐FU/Oxa treatment in the presence of Baf A1, suggesting that FBXW2 was incorporated in autophagosome and degraded through autophagy (Figure S10B).

**FIGURE 6:**
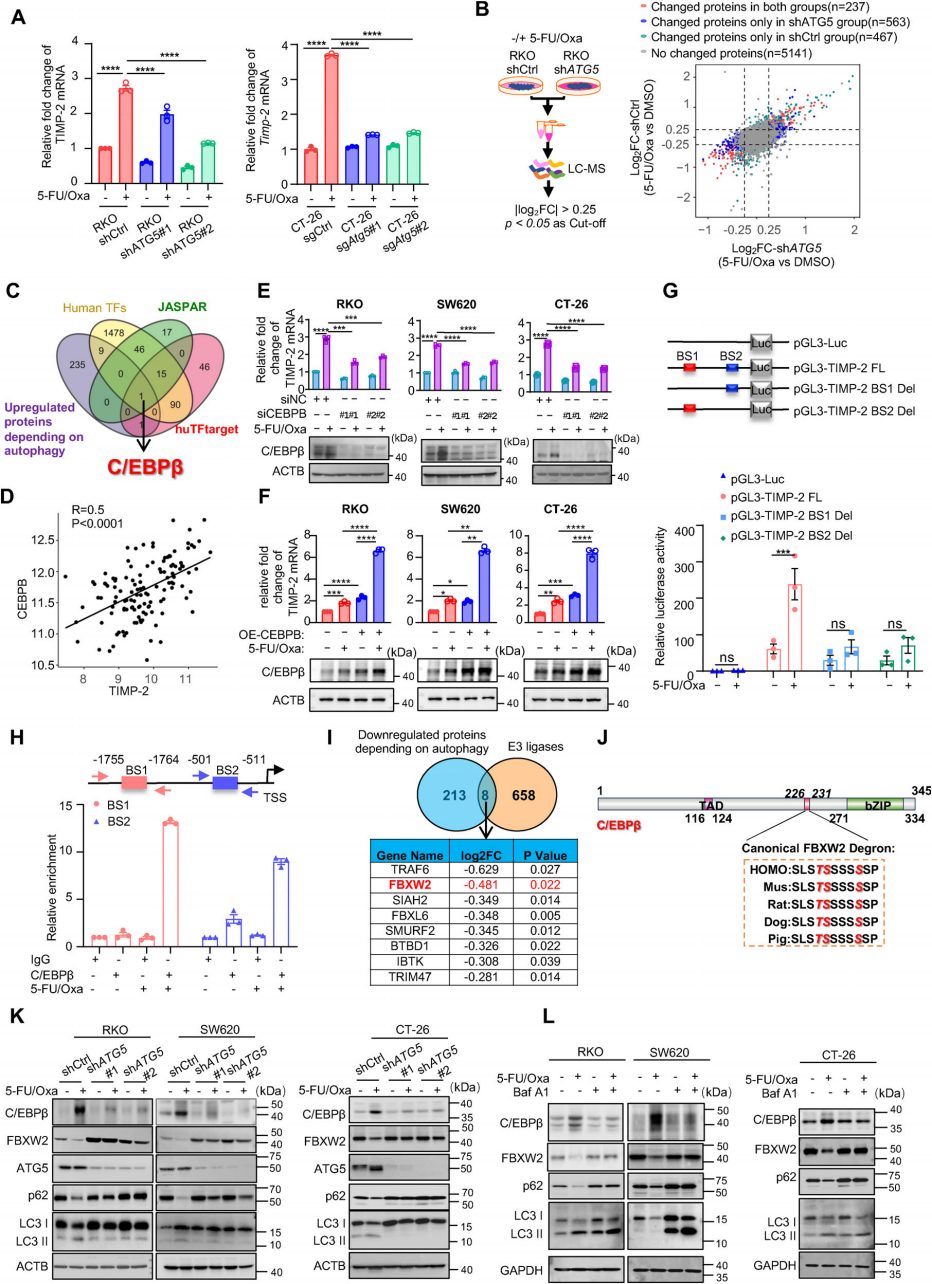
FBXW2‐C/EBPβaxis mediates autophagy‐dependent TIMP‐2 upregulation induced by chemotherapy. (A) qRT‐PCR analysis of TIMP‐2 mRNA expression upon treatment with 5‐FU/Oxa, with or without autophagy inhibition, in RKO and CT‐26 cells. (B) Left: experimental timeline for collecting cell pellets for LC‐MS analysis. Right: scatter plot of differentially expressed proteins (DEPs) between indicated groups. In the shCtrl group alone, a total of 467 DEPs were identified, comprising 246 upregulated and 221 downregulated proteins. (C) Venn diagram depicting the overlap of transcription factors derived from four datasets. (D) Correlation between *CEBPB* and *TIMP‐2* expression in CRC tissues (GSE72970). (E) qRT‐PCR analysis of TIMP‐2 mRNA in RKO, SW620, and CT‐26 cells transfected with scrambled siRNA(si‐Ctrl) or two independent siRNAs targeting CEBPB, with or without 5‐FU/Oxa treatment. CEBPB knockdown efficiency was confirmed by WB. F) qRT‐PCR analysis of TIMP‐2 mRNA in RKO, SW620, and CT‐26 cells overexpressing CEBPB, with or without 5‐FU/Oxa treatment. CEBPB overexpression was confirmed by WB. (G) Top: schematic of luciferase reporter constructs carrying the TIMP‐2 promoter with or without the indicated mutations. Bottom: dual‐luciferase assay showing the relative activity of each promoter mutant in response to 5‐FU/Oxa treatment. (H) Top: schematic depicting the primer design for amplifying distinct regions across the TIMP‐2 promoter. Bottom: quantitative ChIP‐PCR assay measuring C/EBPβ occupancy at those regions in RKO cells treated with or without 5‐FU/Oxa. (I) Venn diagram and summary table showing autophagy‐dependent downregulated E3 ligases induced by 5‐FU/Oxa treatment. (J) Evolutionary conservation of the FBXW2 canonical degradative motif within C/EBPβ. (K) Immunoblot analysis of the indicated antibodies in RKO, SW620, and CT‐26 upon exposure to 5‐FU/Oxa, with or without ATG5 knockdown. (L) Immunoblot analysis of the indicated proteins in RKO, SW620, and CT‐26 cells pretreated with 5‐FU/Oxa for 24 h, followed by ± Bafilomycin A1 (Baf A1, 10 nM) for an additional 12 h before harvest. Data are shown as mean ± SEM. Statistical analysis was performed using TWO‐way ANOVA with multiple comparisons. Significance is indicated as ^*^ *p* < 0.05, ^**^ *p* < 0.01, ^***^ *p* < 0.001, ^****^ *p* < 0.0001, ns, not significant.

### 2.7 FBXW2 Interacts With C/EBPβ and Promotes Its Proteasome dependent‐ Degradation‐

To further clarify how FBXW2 regulates C/EBPβ, molecular docking was performed to predict the binding mode between the two proteins. The results revealed a favorable interaction, with a docking score of <–200 kcal/mol and a confidence score > 0.7 (Figure 7A). Further co‐immunoprecipitation experiments confirmed interaction between FBXW2 and C/EBPβ under both exogenous overexpression and endogenous conditions, even regardless of 5‐FU/Oxa treatment (Figure 7B,C). As an E3 ubiquitin ligase, FBXW2 regulates substrate expression through the ubiquitin‐mediated proteasomal degradation pathway. Indeed, overexpression of FBXW2 led to decrease of C/EBPβ protein, which was blocked by pre‐treatment with MG132, suggesting that FBXW2 regulated C/EBPβ degradation via the ubiquitin proteasome pathway (Figure 7D). Consistently, knockdown of FBXW2 promoted C/EBPβ expression and restoration of FBXW2 suppressed its upregulation, whereas addition of MG132 substantially abrogated this effect (Figure 7E). Furthermore, overexpression of FBXW2 decreased C/EBPβ protein stability. By contrast, silencing of FBXW2 profoundly extended the half‐life time of C/EBPβ protein (Figure 7F,G). More importantly, overexpression of FBXW2 significantly enhanced ubiquitination level of C/EBPβ, whereas FBXW2‐ΔF mutant (deletion of F‐box), which lacked the ability to recruit other components of SCF ubiquitin ligase complex, had little effect [46, 47, 48] (Figure 7H). The classical ubiquitination pathway includes K48‐ and K63‐linked ubiquitination, and that K48‐linked ubiquitination is the key route mediating substrate entry into proteasomal degradation. Our co‐IP results showed that the K48R mutation significantly suppressed FBXW2‐induced ubiquitination of C/EBPβ, whereas the K63R mutation caused no significant change, further suggesting that FBXW2 promotes ubiquitin‐mediated proteasomal degradation of C/EBPβ (Figure 7I). Further analysis of the FBXW2/ C/EBPβbinding model displayed that the predicted amino acid residues of FBXW2 interacting with C/EBPβare mainly located in the WD domain, which is the key structural domain for FBXW2 to bind its substrates as an E3 ubiquitin ligase. Interestingly, three lysine residues, K302, K315, and K316, are located in proximity to the FBXW2/ C/EBPβinteraction interface (Figure 7J). To identify the specific lysine targeted for ubiquitination, we introduced point mutations at these residues and observed that K to R substitutions at positions 302 and 316 markedly reduced C/EBPβubiquitination, indicating that FBXW2 mediates ubiquitination of C/EBPβ through K302 and K316, consequently modulating its protein stability(Figure 7K). Collectively, these results suggested that FBXW2 interacted and promoted degradation of C/EBPβ through the ubiquitin‐mediated proteasomal pathway.

**FIGURE 7:**
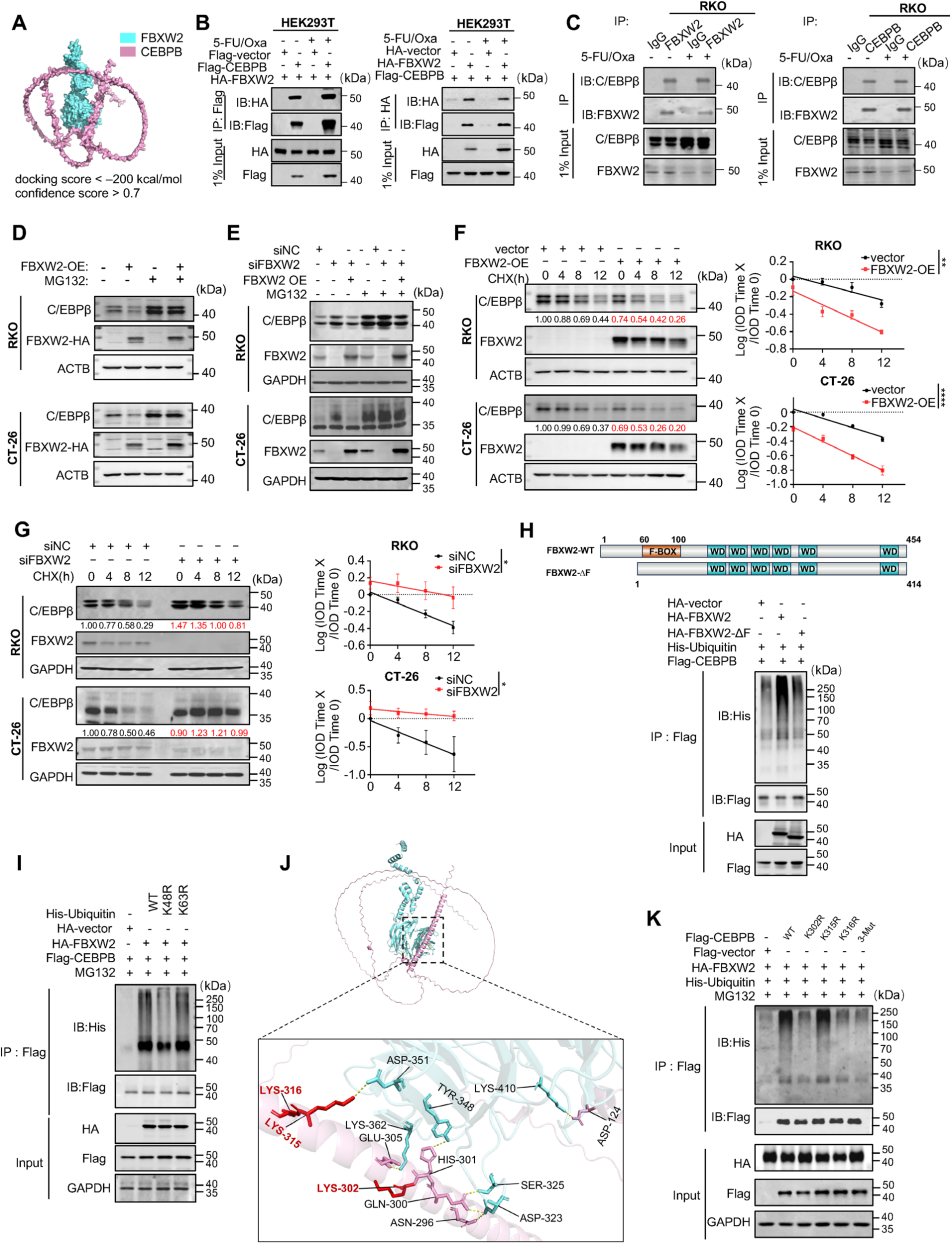
FBXW2 interacts with C/EBPβ and promotes its degradation through ubiquitin proteasome pathway. (A) The computational modeling of FBXW2 and C/EBPβ interaction was performed by AlphaFold3, followed by visualization with PyMOL. Proteins color assignment: CEBPB (Purple), FBXW2 (Blue). (B) Exogenous interaction between C/EBPβ and FBXW2 was examined by co‐immunoprecipitation (Co‐IP) in HEK293T cells transfected with the indicated plasmids, with or without 5‐FU/Oxa treatment. Immunoprecipitation was performed with anti‐Flag or anti‐HA antibodies. (C) Co‐IP analysis of endogenous interaction between C/EBPβ and FBXW2 upon treatment with 5‐FU/Oxa or not. Immunoprecipitation was performed using antibody against FBXW2 or C/EBPβ. (D) Immunoblot analysis of C/EBPβ expression in RKO and CT‐26 cells overexpressing FBXW2, with or without proteasome inhibitor MG132 (20 µm, 6 h) treatment. (E) Immunoblot analysis of C/EBPβ expression in RKO and CT‐26 cells with FBXW2 knockdown or rescue, in the presence or absence of proteasome inhibitor MG132. (F,G) Protein stability of C/EBPβ was assessed by cycloheximide (CHX,10ug/ml) chasing experiment in RKO and CT‐26 cells with FBXW2 overexpression or knockdown, followed by calculation of protein degradation kinetics. (H) HEK293T cells were co‐transfected with Flag‐CEBPB, His‐Ubiquitin, and HA‐tagged wild‐type FBXW2 (FBXW2‐WT) or its F‐box deletion mutant (FBXW2‐∆F). C/EBPβ ubiquitination was assessed by immunoprecipitation with anti‐Flag antibody followed by immunoblotting with anti‐His antibody. (I) HEK293T cells were co‐transfected with Flag‐CEBPB, HA‐FBXW2, and His‐tagged wild type ubiquitin (WT)or mutants with K‐48‐R(K48R) or K‐63‐R(K63R) substitution. Immunoprecipitation was carried out using anti‐Flag antibody, and C/EBPβ ubiquitination was evaluated by immunoblotting with anti‐His antibody. (J) Computational modeling of FBXW2, and C/EBPβ interaction surface was performed by AlphaFold3, followed by visualization with PyMOL. FBXW2 residues are rendered in blue, whereas C/EBPβ residues are colored in red (specifically K302, K315, and K316) and pink (remaining residues). (K) HEK293T cells were co‐transfected with HA‐FBXW2, His‐Ubiquitin and Flag‐tagged CEBPB with the indicated K to R substitution. Immunoprecipitation was performed using anti‐Flag antibody, and C/EBPβ ubiquitination was evaluated by immunoblotting with anti‐His antibody.

### 2.8 Autophagy Exhibits Positive Correlation With TIMP‐2 Expression and Associated with Chemo‐Sensitivity in Clinic

To evaluate the clinical relevance of autophagy‐TIMP‐2 axis, we analyzed public CRC datasets. Autophagy score exhibited a strong positive correlation with *TIMP‐2* in three independent datasets, including TCGA_COADREAD, GSE45404 (FOLFOX pre‐treatment), GSE69657 (FOLFOX post‐treatment), supporting autophagy‐mediated transcriptional regulation of TIMP‐2 irrespective of chemotherapy [51, 52, 53] (Figure 8A–C). Additionally, patients with higher sensitivity to 5‐FU based chemotherapy also displayed higher *TIMP‐2*, highlighting the clinical relevance of autophagy‐TIMP‐2 axis to sensitivity of 5‐FU based chemotherapy in CRC patients (Figure 8D,E). Moreover, we performed multiplex immunofluorescence co‐staining with p‐ATG16L1, TIMP‐2, GZMB, and PanCK in typical responder and non‐responder of CRC patients to 5‐FU based chemotherapy. The images indicated that the responder displayed higher expression of p‐ATG16L1 and TIMP‐2 in the tumor region accompanied by increased presence of GZMB within the stromal area, compared to the non‐responder (Figure 8F). We further analyzed p‐ATG16L1 puncta formation, TIMP‐2 expression, and GZMB^+^ T‐cell frequency in a cohort of clinical samples from the Sixth Affiliated Hospital of Sun Yat‐sen University, collected after neoadjuvant chemotherapy. Patient clinicopathological characteristics are detailed in Table S1. Similar to the above findings, p‐ATG16L1 puncta formation, the marker representing autophagy induction, displayed strongly positive correlation with TIMP‐2 expression, which exhibited positive correlation with active anti‐tumor immunity markers like GZMB (Figure 8G,H; Figure S11). Patients with p‐ATG16L1^high^/TIMP‐2^high^ expression constituted a significantly higher proportion of the responder group, whereas those with p‐ATG16L1^low^/TIMP‐2^low^ expression were markedly enriched in the non‐responder group (Figure 8I). In line with this, a higher frequency of patients with elevated LC3B puncta levels was observed in the responder group, whereas non‐responders showed the opposite pattern (Figure 8J; Figure S12A). To further expand the clinical significance of our study, we corroborated our findings using data from two distinct clinical cohorts (GSE24550 and GSE103479) and found that patients with higher expression of TIMP‐2 and ATG16L1 had significantly better disease‑free survival (DFS) and overall survival (OS) than those with lower expression (Figure S12B,C). Collectively, these data reinforced the clinical significance of the newly unlocked autophagy‐TIMP‐2 axis in modulating chemotherapeutic response in CRC patients. p‐ATG16L1^high^ TIMP‐2^high^ patients also accounted for an obviously higher percentage in the responder group, whilst the proportion of p‐ATG16L1^low^ TIMP‐2^low^patients was much higher in the non‐responder group

**FIGURE 8:**
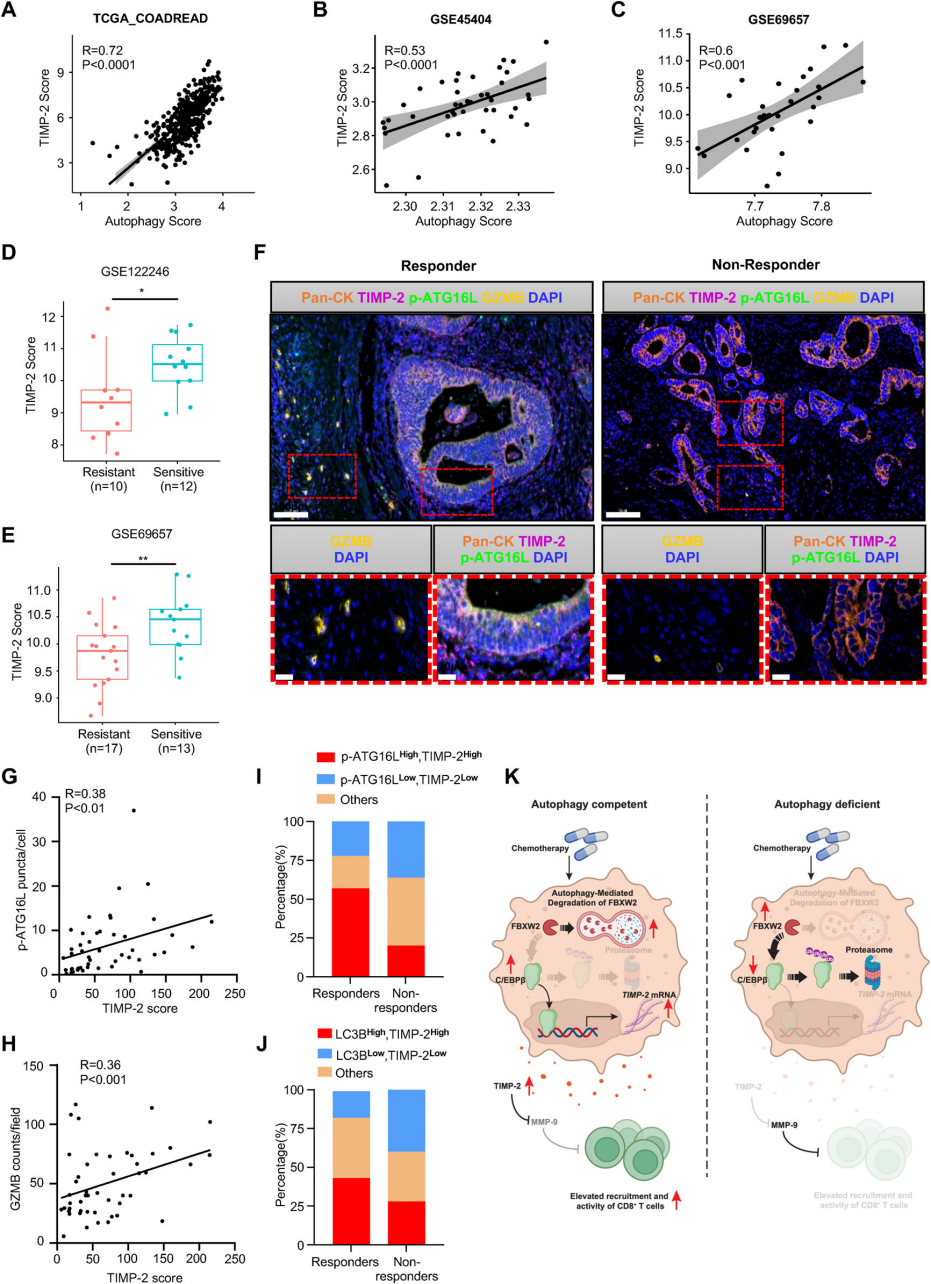
Clinical relevance of the autophagy‐TIMP‐2 axis in CRC patients. (A–C) Correlation between TIMP‐2 expression and autophagy score. (D,E) TIMP‐2 expression levels in resistant versus sensitive CRC patients treated with 5‐FU‐based chemotherapy, derived from two independent cohorts. (F) Representative multiplex immunofluorescence images of responder and non‐responder patient samples, co‐stained for Pan‐CK (orange), TIMP‐2 (pink), p‐ATG16L1 (green), and GZMB (gold). (G) Correlation between TIMP‐2 expression and p‐ATG16L1 puncta per cell, indicating autophagy activity, in the SYSU‐the sixth Affiliated Hospital cohort (n = 48). (H) Correlation between TIMP‐2 expression and GZMB counts per field, indicating active anti‐tumor immunity, in the same cohort as in (G). (I) Patients were stratified into responders and non‐responders based on the tumor regression grade (TRG) score, with TRG2 defined as responder (n = 23) and TRG3 (n = 25) as non‐responder. Proportions of patients with p‐ATG16L1^High^/TIMP‐2^High^; p‐ATG16L1^Low^/TIMP‐2^Low^ or others were analyzed and shown in the graph. (J) Proportions of patients with high or low expression of LC3‐II puncta, and others were analyzed and depicted in the graph, comparing responders and non‐responders. (K) Schematic diagram illustrating tumor cell‐intrinsic autophagy acts via the FBXW2/C/EBPβ/TIMP‐2 axis to regulate the TIME and chemosensitivity.

## 3 Discussion

Chemotherapies like FOLFOX still remain the first‐line standard regimens for CRC, particularly in MSS/MSI‐L patients who account for the majority of cases. Accumulating evidence indicates that chemotherapeutic agents exert immunomodulatory effects that substantially influence clinical outcomes. Chemotherapy‐mediated clearance of MDSCs and polarization of macrophages toward an M1 phenotype alleviate the immunosuppressive tumor microenvironment, concurrently with the activation of CD8^+^ T cells induced by chemotherapy‐elicited immunogenic cell death (ICD). In multiple cancer types, patients with higher infiltration of activated CD8^+^ T cells and M1 macrophages tend to have better prognosis following chemotherapy. Specifically, in colorectal cancer (CRC), elevated levels of CD8^+^ effector T cells expressing activation markers such as CD69 and IFN‐γ are associated with improved therapeutic response [54, 55]. Although chemotherapy‐induced ICD has been considered as an essential route to boost anti‐tumor immune response, its efficacy is frequently limited in CRC by genetic alteration of ICD‐associated signaling axes, such as p53 mutations [56, 57]. Therefore, other potential signaling pathways that regulate the antitumor immune response remain to be further elucidated. Previous literatures have illustrated complicated roles of autophagy within immune cells, which was largely determined by the downstream inflammation associated networks [37, 58, 59]. In CRC, the role of autophagy in modulating sensitivity of tumor cells to chemotherapy in vitro or xenograft mouse model has been broadly investigated and commonly considered to confer resistance. However, regarding the intimate dialogue between tumor cells and TIME, it remains to be elucidated that how does tumor cell‐intrinsic autophagy impact the TIME, thus influencing the treatment outcome. In this study, using CyTOF to profile the alteration of TIME upon exposure to 5‐FU/Oxa with or without autophagy inhibition, we have identified an unrecognized role of tumor cell‐intrinsic autophagy in manipulating anti‐tumor immune response, therefore enhancing chemo‐sensitivity. Further cytokines array analysis demonstrated that TIMP‐2 was the key downstream effector regulated by autophagy. TIMP‐2 KO mimicked the phenotype of autophagy inhibition, while ectopic expression of TIMP‐2 in autophagy deficient tumor cells could effectively rescue sensitivity to 5‐FU/Oxa in murine models, which were also accompanied by an active TIME. More importantly, our analysis of human CRC tumor tissues indeed showed a consistent pattern. Autophagy status and TIMP‐2 expression exhibited a positive correlation with anti‐tumor immunity and the clinical response to chemotherapy.

Autophagy is a conserved degradative process within the cell, which helps to recycle proteins or sub organelles for maintaining cellular homeostasis. Abnormal autophagic protein degradation has been implicated in cancer either as a foe or a friend. Autophagy‐driven PD‐L1 degradation was considered as an essential reason causing resistance to immune checkpoint blockade in hepatocellular carcinoma (HCC). Similarly, MHC‐I/II degradation through autophagy impaired antigen presentation and promoted immune evasion in multiple cancers, including PDAC, lung cancer, and breast cancer. On the contrary, a recent seminal study reported that KRAS protein in PDAC could be degraded through autophagy, which provided an alternative strategy to target KRAS via induction of autophagy [60, 61, 62, 63, 64]. Leveraging in silico prediction and experimental validation, we identified that the E3 ligase FBXW2 could be a novel cargo that can be degraded through autophagy upon treatment with 5‐FU/Oxa. Reduction of FBXW2 reciprocally led to accumulation of its potential substrate‐C/EBPΒ, therefore transcriptionally upregulating TIMP‐2.

TIMP‐2, as an endogenous inhibitor of MMP‐2/9, has elicited ambiguous functions in a context‐dependent manner. For example, TIMP‐2 can suppress tumor growth and metastasis across diverse cancers such as breast cancer lung cancer, and glioma, whereas it has also been implicated in promoting proliferation, invasion, and chemo‐resistance in ovarian cancer. However, it is noteworthy that current research on how TIMP‐2 regulates tumor progression remains relatively preliminary, and the underlying mechanisms are not yet clearly understood. Through the construction of wild‐type or loss‐of‐function TIMP‐2 mutants combined with mouse model experiments, we showed that TIMP‐2 could enhance immune response and chemo‐sensitivity in a manner dependent on its inhibition of MMP‐2/9 activity. Co‐administration of MMP‐2/9 inhibitor or exogenous delivery of TIMP‐2 was efficient to restore sensitivity of autophagy‐deficient CRC mouse model to 5‐FU/Oxa.

In summary, our study not only unraveled a novel role of autophagy in modulating anti‐tumor immune response provoked by 5‐FU/Oxa through FBXW2/ C/EBPβ/TIMP‐2 axis but also provided potential combination therapeutic strategies, including targeting MMP‐2/9 or artificially induction of TIMP‐2 for those CRC patients with compromised autophagy to enhance chemo‐sensitivity (Figure 8K).

## 4 Materials and Methods

### 4.1 Cell Culture and Reagents

The human embryonic kidney HEK 293T cell line, human colorectal cancer (CRC) cell lines (including RKO, HCT116, SW620), and the mouse CRC CT26 cells were purchased from the American Type Culture Collection (ATCC). HEK 293T, RKO, SW620 cells were cultured in DMEM, while HCT116 and CT26 cells were maintained in RPMI 1640 medium supplemented with 10% fetal bovine serum (FBS) and 1% Penicillin/Streptomycin (PS). All cell lines were maintained at 37°C in a humidified incubator with 5% CO_2_. Reagents used in this study are listed in Table S2.

### 4.2 Small Interfering RNA Silencing

Two independent siRNAs targeting *CEBPB* or *FBXW2* (GenePharma, China) were transfected into RKO and SW620 cells with or without autophagy inhibition using RNAiMAX (#13778030; Thermo Fisher, USA) according to the manufacturer's instructions. Scramble oligos were used as control. After 24 h, cells were treated with 5‐FU/Oxa or not and subjected to further experiments. The siRNA sequences are listed in Table S3.

### 4.3 Generation of Stable Cell Lines

To establish stable cell lines with silencing or overexpression of targeted genes, guide RNAs and shRNAs targeting the relevant genes or cDNA were cloned into Lenti‐CRISPR‐V2‐Puro, pLKO.1‐puro, or PMN‐puro empty vector, respectively. The virus particles were produced using HEK 293T cells with the help of packaging plasmids or Plat‐A cells described previously [65]. The virus particles were collected by centrifugation at 1000 g for 5 min, followed by infiltration through a 0.45‐µm filter. CT‐26, RKO, and SW620 cells were grown to 60% confluency and incubated with virus particles for 48 h, followed by puromycin selection for another 72 h. The sgRNA or shRNA sequences and primers for TIMP‐2 mutant construction are listed in Tables S3 and S4, respectively.

### 4.4 Animal Studies

All studies were carried out in accordance with the recommendations in the Guide for the Care and Use of Laboratory Animals approved by the Ethics Institutional Review Boards of the Sixth Affiliated Hospital of Sun Yat‐sen University. Six to eight weeks female immunocompetent BALB/c mice or immune‐deficient Nude mice were purchased from Gempharmatech Co., Ltd. (China) and cultured in specific pathogen‐free facilities. 2 × 10^6^ CT26 cells were subcutaneously injected into BALB/c mice or Nude mice to evaluate tumor growth. Tumor volume was measured with a digital caliper and calculated as width (in mm)^2^ × length (in mm)/2. Drugs were administrated intraperitoneally following the stated regimens: 5‐FU (25 mg/kg) plus oxaliplatin (2.5 mg/kg) every other day for 2 weeks; chloroquine (CQ, 60 mg/kg) or SB‐3CT (10 mg/kg) daily. For CD8^+^ T depletion, neutralizing CD8 antibody (#CP128, BioXCell, 100 ug/mouse) was given intraperitoneally at the beginning of tumor inoculation, followed by subsequent doses every 3 days thereafter.

### 4.5 CyTOF Analysis

Freshly harvested tumors were cut into small pieces (∼1 mm3) with scissors and digested with collagenase IV (#V900893, VETEC) for 1 h at 37°C. Dissociated tumor samples were homogenized and filtered through a 70‐µm strainer to obtain a single‐cell solution. ACK Lysis Buffer (#BS‐01‐05, PLT) was utilized to lyse erythrocytes. Then cells were stained with Cell‐ID Cisplatin‐194Pt (#201194, FLUIDIGM) for 5 min, followed by incubation with blocking solution for 20 min on ice. Cells were stained with antibody mix (#201300, FLUIDIGM) for 30 min on ice for surface markers. For intracellular marker staining, cells were treated with permeabilization buffer (#00‐8333‐56, Thermo Fisher) according to the manufacturer's instructions, followed by antibody mix staining for a further 30 min. After being washed and resuspended with deionized water, 20% EQ beads (Fluidigm) were added before examined by a Helios3 CyTOF system (Fluidigm, USA). After normalization using a method based on the EQ Four Element Calibration Beads, the CyTOF data were analyzed using the Cytobank platform(https://www.cytobank.org/) and the Flowjo software.

Cells were stained with 37 antibodies including: CD45, CD3e, TCRb, CD4, CD8a, CD44, CD62L, CD25, CD127, FoxP3, CD183 (CXCR3), CD194 (CCR4), CD196 (CCR6), CD69, TIM‐3, CTLA4, LAG‐3, Granzyme B, CD103, CD278 (ICOS), CD279 (PD1), CD19, B220, NKp46, CD49b, CD11b, CD11c, MHCII, Ly6C, Ly6G, Gr1, F4/80, CD80, CD86, CD206, iNOS, Ki67. The major immune cell subsets were classified according to the following marker combinations:NK cells:CD45^+^ CD3^−^ CD19^−^ NKP46^+^ CD49b^+^;CD8^+^ T cells:CD45^+^ CD3^+^ TCRb^+^ CD8^+^ CD4^−^;CD4^+^ T cells:CD45^+^ CD3^+^ TCRb^+^ CD4^+^ CD8^−^; DPT (Double Positive) cells: CD45^+^ CD3^+^ TCRb^+^ CD4^+^ CD8^+^; gdT cells: CD45^+^ CD3^+^ TCRb^−^; B cells: CD45^+^ CD3^−^CD19^+^; MDSC: CD45^+^ CD3^−^ CD11b^+^ Gr1^+^; DC cells: CD45^+^ CD3^−^ CD11C^+^ MHCII^+^; Macrophage: CD45^+^ CD3^−^ CD11b^+^ F4/80^+^; Granulocyte: CD45^+^ CD3^−^ CD11b^+^.

### 4.6 Cytokine Antibody Array and ELISA Assay

For cytokine antibody array, RKO cells with or without autophagy inhibition were treated with 5‐FU/Oxa for 48 h or not. Cell culture supernatants were collected and used directly without further dilution. Semiquantitative detection of 80 human cytokines and chemokines were performed using the RayBio C‐series Human Cytokine Antibody Array C5 (#AAH‐CYT‐5‐8, RayBiotech) according to the manufacturer's instructions. Detection of dots was performed with the ChemiDoc MP Imaging Systems (Bio‐Rad). The TIMP‐2 levels in supernatants from both RKO and CT‐26 with or without autophagy inhibition upon exposure to 5‐FU/Oxa were assessed using ELISA assay kit according to the manufacturer's protocol.

### 4.7 RNA Extraction and qRT‐PCR

Total RNA was extracted using TRIzol (#15596018,Thermo Fisher) or RNeasy Mini kit(#LS1040, Promega) according to manufacturer's instruction. cDNA was reversely transcribed and subjected to PCR amplification using SYBR Green (#1725125; Bio‐rad, USA) on a Light Cycler 480 instrument (Roche, Basel, Switzerland). Relative expressions of each gene were normalized to Actin. Primers used for qRT–PCR are summarized in Table S4.

**CUT&Tag‐qPCR** RKO cells were treated with 5‐FU/Oxa or not and subjected to qPCR assay after CUT&Tag experiment (#N258‐YH01, Novoprotein). Briefly, cells were bound to concanavalin A‐coated magnetic beads. The beads‐bound cells were then incubated with C/EBPβ antibody (#sc‐7962, Santa Cruz) at a 1:100 dilution overnight at 4°C. After that, the cells were washed with buffer containing 0.05% digitonin and subsequently incubated with transposase for 1 h. Cleavage was initiated by adding 1 µL of 100 mM MgCl_2_ and incubating for 1 h. The reaction was stopped by adding the quenching buffer. The supernatant, containing the released C/EBPβ‐DNA complexes, was collected by centrifugation, purified, and subsequently analyzed by qPCR. The Primer sequences are provided in Table S4.

### 4.8 Dual‐Luciferase Reporter Assay

Dual‐luciferase reporter assay was performed using RKO cells treated with 5‐FU/Oxa or not. The cells were seeded in a 24‐well plate and transfected with plasmids as follows: pGL3 empty vector (pGL3‐Luc); full‐length TIMP‐2 promoter containing two potential C/EBPΒ binding site‐BS1 and BS2 (pGL3‐TIMP‐2 FL); promoter region of TIMP‐2 with either BS1 or BS2 deletion (pGL3‐TIMP‐2 BS1 Del or pGL3‐TIMP‐2 BS2 Del). pRL null was used as an internal control and co‐transfected with the indicated plasmids at a ratio of 1:100. 24 h after transfection, cells were treated with 5‐FU/Oxa for additional 24 h or not. The luciferase activity was measured using Dual‐Luciferase Reporter Assay System Kit (#E1910, Promega) according to the manufacturer's instructions. Primers for amplifying promoter regions of TIMP‐2 are shown in Table S4.

### 4.9 Co‐IP and Immunoblotting

Western blot analysis and immunoprecipitation were performed described previously [66]. For co‐immunoprecipitation assay, 293 T cells expressing indicated tagged proteins were lysed in IP lysis buffer (#89900, Thermo Fisher) containing protease and phosphatase inhibitor cocktails. The cell lysates were incubated with Flag or HA antibody at 4°C overnight, followed by immobilization using Protein A agarose (#11134515001, Sigma) at 4°C for further 4 h. The agarose beads were washed three times with washing buffer. The precipitated proteins were boiled in 1× SDS loading buffer and subjected to immunoblotting analysis. The antibodies used are provided in Table S5.

### 4.10 Flow Cytometry

Single‐cell suspensions were prepared from tumor tissues for flow cytometric analysis. Briefly, tumors were minced with surgical scissors and enzymatically dissociated in a buffer containing Collagenase Type IV (Stem Cell Technologies, #07909) and hyaluronidase buffer (Stem Cell Technologies, #07912), 20 µg/mL DNase I (Stem Cell Technologies, #07900), and 10 mM HEPES in 5% FBS, for 40 min at 37 °C with constant agitation. The cell suspension was filtered through a 70‐µm cell strainer and then subjected to red blood cell lysis (Miltenyi, #130‐094‐183). For immunostaining, cells were first washed and incubated with surface markers. They were then incubated with BD Horizon Fixable Viability Stain 520 (BD, #564407) to distinguish viable from non‐viable cells. Subsequently, cells were fixed and permeabilized using a commercial Fixation/Permeabilization kit (Thermo Fisher Scientific, 00‐5523‐00): cells were resuspended in a 1:3 mixture of Fixation/Permeabilization Concentrate and Diluent for 1 h at room temperature. After fixation, cells were washed and resuspended in permeabilization buffer for intracellular staining. Flow cytometry data were acquired on a Beckman CytoExpert flow cytometer and analyzed using FlowJo software (BD Biosciences). The antibodies used are listed in Table S5.

### 4.11 Statistical Analysis

All the statistical analyses were performed using GraphPad Prism10 and presented as mean ± standard error (SEM). Image J was used to analyze the localization between the two stained proteins. Student's t test and the nonparametric test were used to assess differences between two groups. One‐way or Two‐way ANOVA with multiple comparison test was used to assess the statistical significance for the experiments with >2 independent groups. Data with error bars are shown as mean ± SEM. ns, no significance, ^*^ *p* < 0.05, ^**^ *p* < 0.01, ^***^ *p* < 0.001, ^****^ *p* < 0.0001.

## Author Contributions

**Wenyu Wang** and **Bing Cheng** conceived the study and prepared the manuscript. **Bing Cheng** and **Chaoying Wei** performed most of the experiments and data interpretation. **Zhitong Niu** did the bioinformatic analysis and some cell experiments. **Jinxin Chen**, **Guojie Long**, **Yingzhen Weng**, **Qiufeng Liu**, and **Yue Jiang** contributed to the technical assistance and analysis. **Panpan Wang**, **Ronghua Zhang**, **Xiaojian Wu**, and **Qiang Yu** provided crucial resources or conceptual advice. **Bing Cheng** and **Wenyu Wang** wrote the manuscript with input from other co‐authors.

## Conflicts of Interest

Cheng,B, Wang, W., Wei, C., and Niu,Z. are inventors of patent applications related to the technology described in this paper. The other authors declare no competing interests.

## Supporting information




**Supporting File 1**: advs77349‐sup‐0001‐SuppMat.pdf.

**Supporting File 2**: advs77349‐sup‐0002‐SuppMat.docx.

## Data Availability

The data that support the findings of this study are available in Gene Expression Omnibus at https://www.ncbi.nlm.nih.gov/geo/, reference number GSE69657, GSE45404, GSE12246. These data were derived from the following resources available in the public domain: ‐ GSE69657, https://www.ncbi.nlm.nih.gov/geo/query/acc.cgi?acc=GSE69657, ‐ GSE45404, https://www.ncbi.nlm.nih.gov/geo/query/acc.cgi?acc=GSE45404, ‐ GSE12246, https://www.ncbi.nlm.nih.gov/geo/query/acc.cgi?acc=GSE12246
