# Supplementary material for: Autophagy Orchestrates Anti‐Tumor Immunity to Enhance Chemosensitivity via the FBXW2/C/EBPβ/TIMP‐2 Axis in Colorectal Cancer
Source: Adv Sci (Weinh). 2026 Aug 21;13(63):e77349. doi: 10.1002/advs.77349 (PMC13496286; doi:10.1002/advs.77349)

Supplementary Figure 1. Chemotherapy induces autophagy in vitro and in vivo

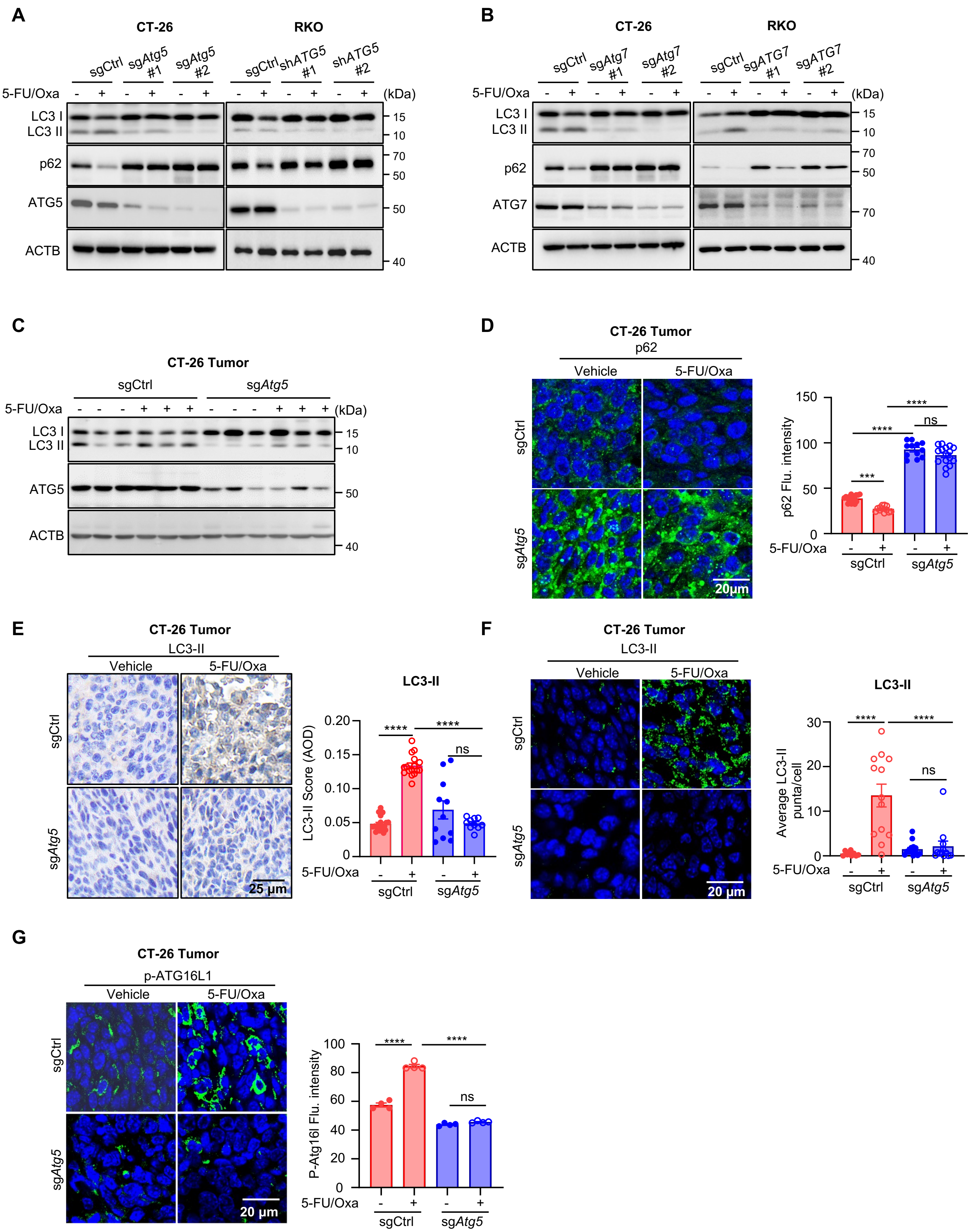

Supplementary Figure 2. CQ phenocopies the effect of Atg5 knockout in reducing chemosensitivity, while exhibiting no systemic toxicity.

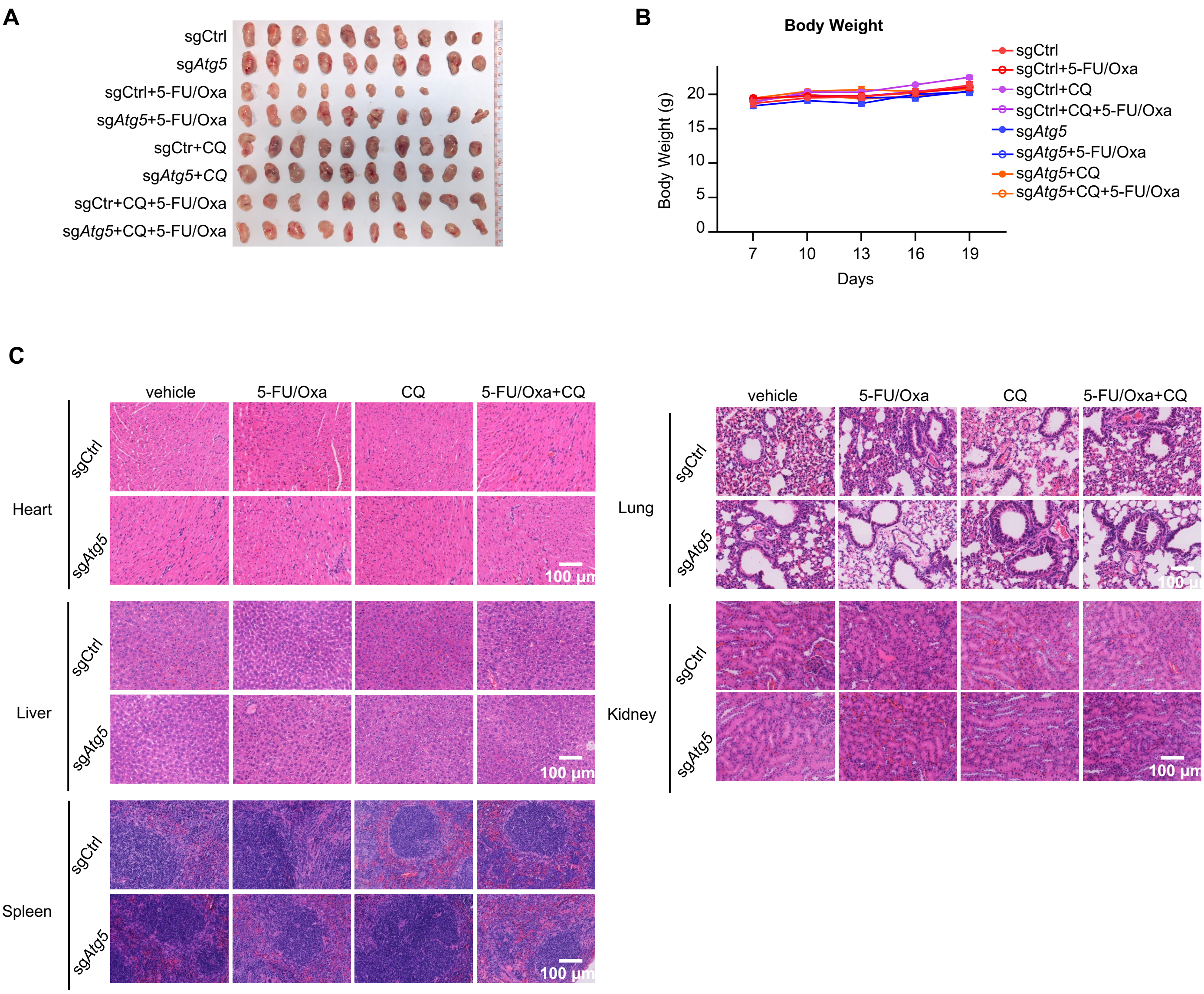

Supplementary Figure 3. CyTOF profiling of the tumor immune microenvironment in response to chemotherapy, with or without autophagy inhibition

A

B

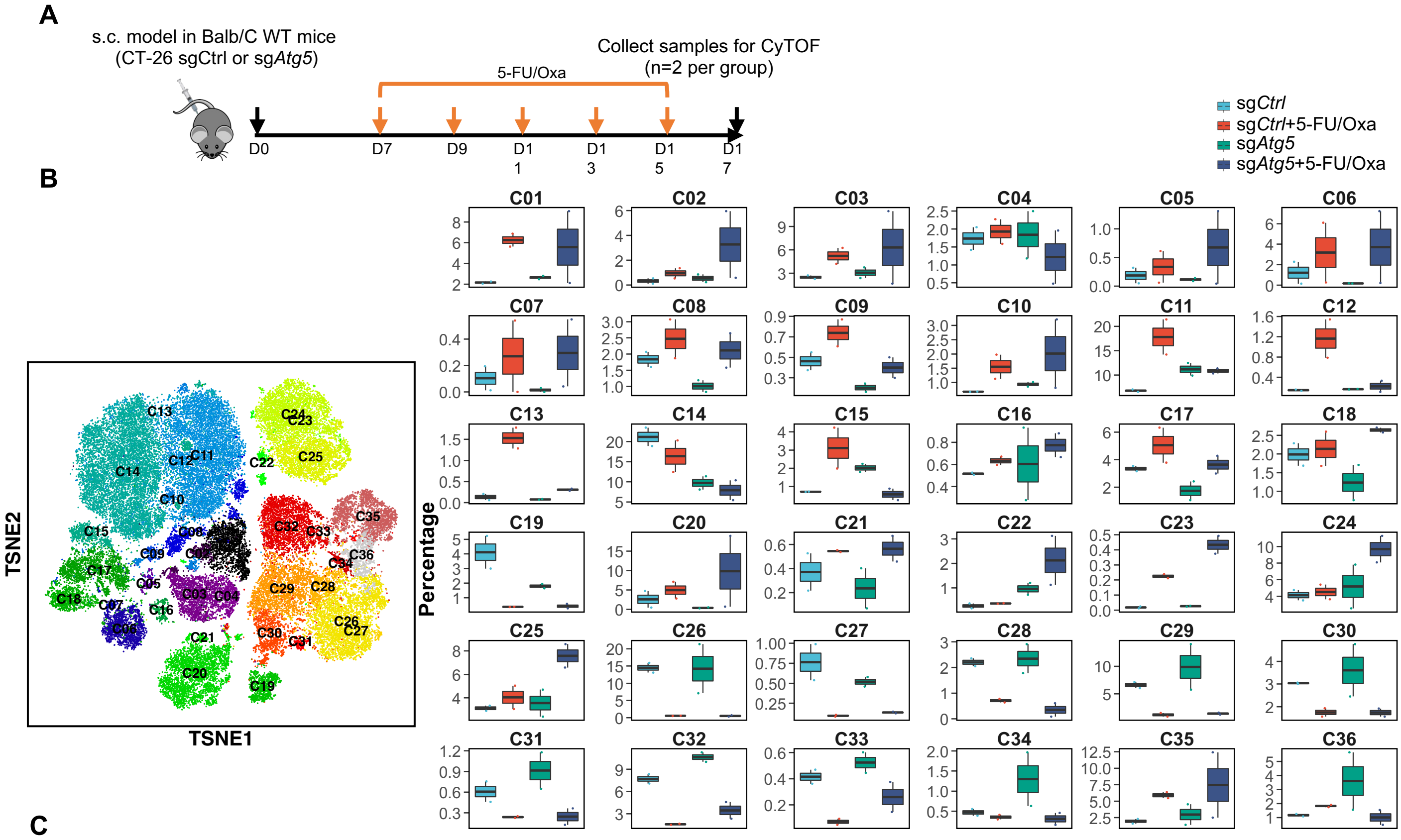

C

Definition for subtypes of immune cells	Clusters
CD8⁺ T	C05,C08,C10-C14
CD4⁺ T	C06-C07,C09,C17-C18
gdT	C16
DPT	C15
NK cells	C01,C03-C04
B cells	C20-C21
MDSC	C22-C25
Macrophage	C26-C27,C29-C33
Granulocyte	C35
DC cells	C02,C19,C28

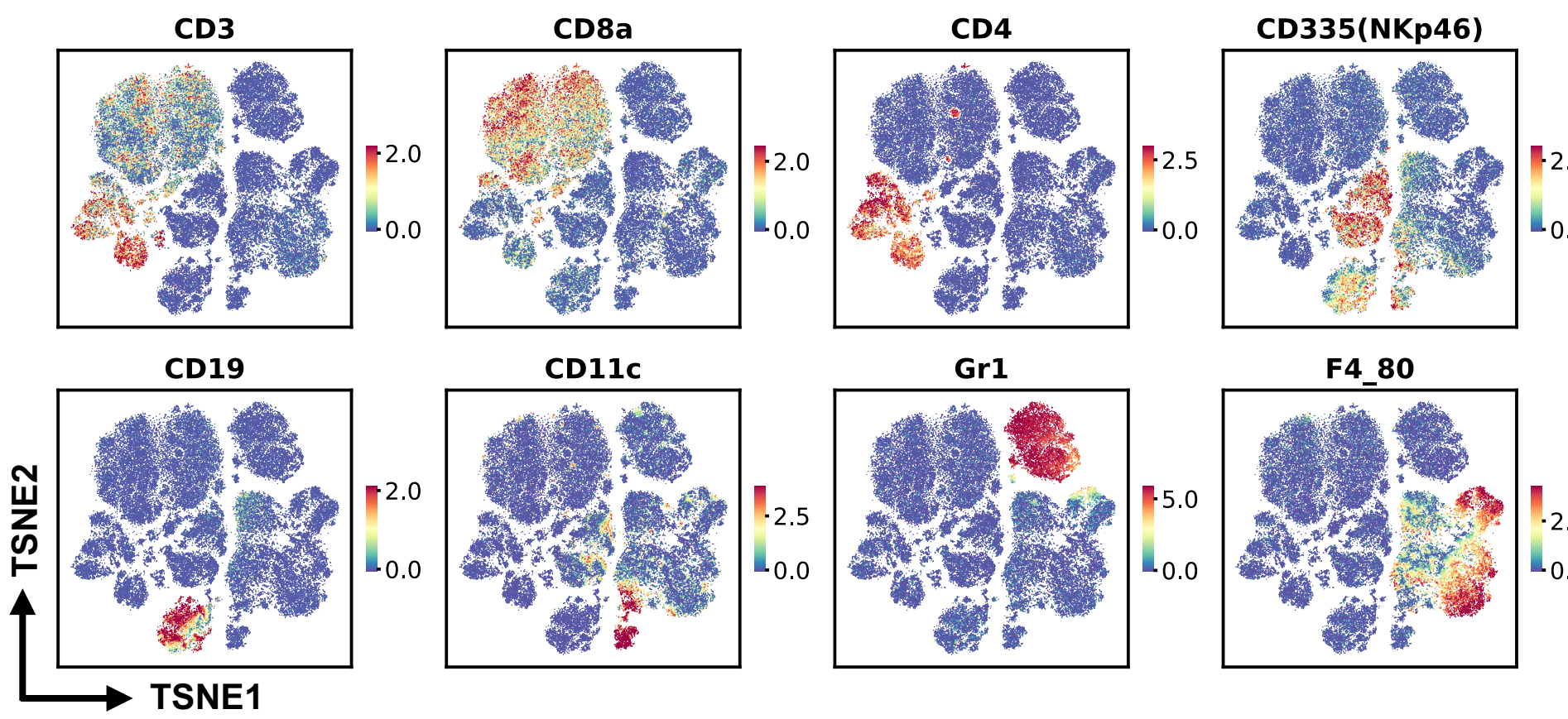

D

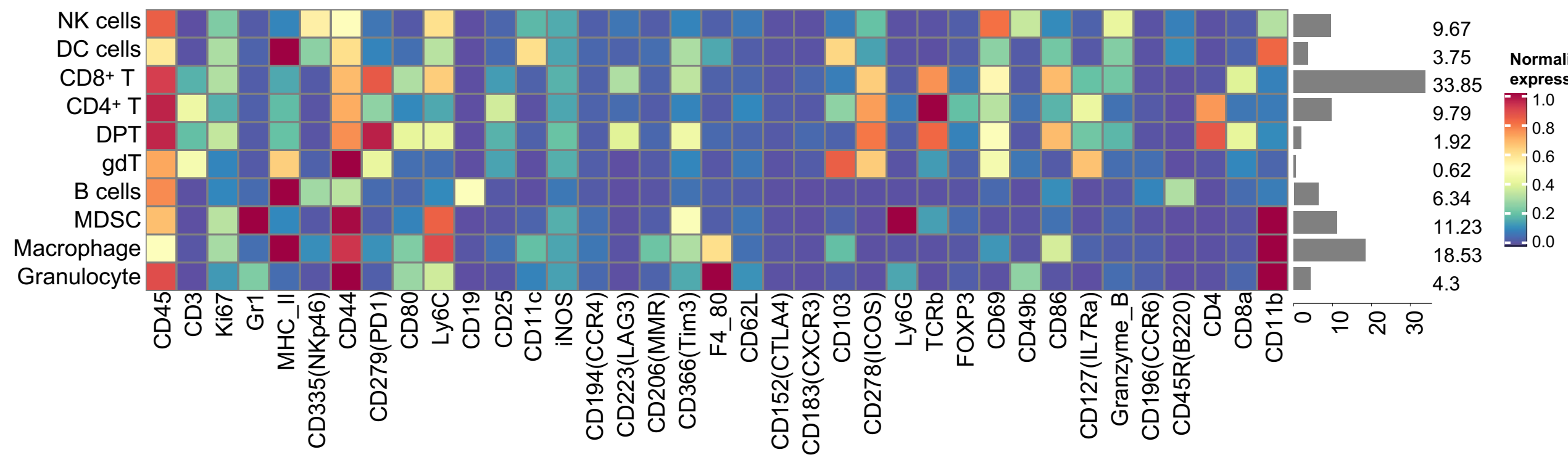

E

F

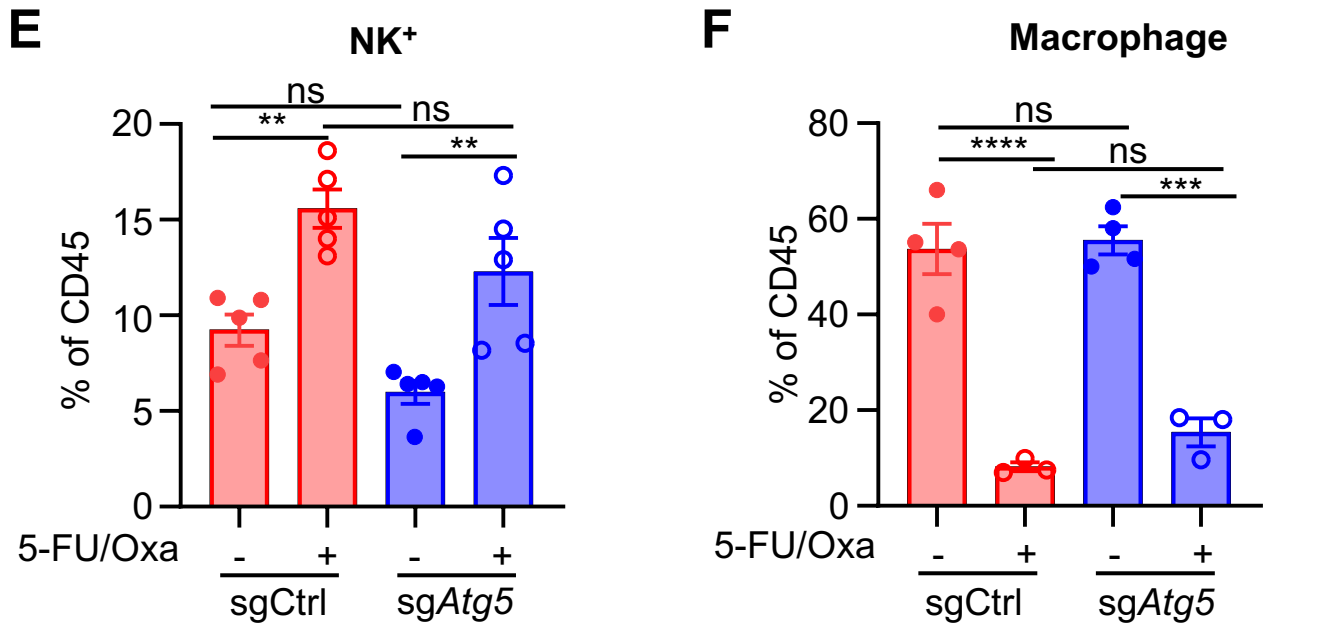

Supplementary Figure 4. Functional annotation of $CD8^+$ and $CD4^+$ T-cell clusters

A

Definition for subtypes of $CD8^+$ and $CD4^+$ T cells	Clusters
Naïve $CD8^+$ T	C05
$Ly6C^+$ $CD8^+$ Tem	C08,C11-C13
$Ly6C^-$ $CD8^+$ Tem	C14
$Ly6C^+$ $CD8^+$ Tcm	C10
Naïve $CD4^+$ T	C06
Treg	C07,C18
$ICOS^+PD1^+$ $CD4^+T$	C09,C17

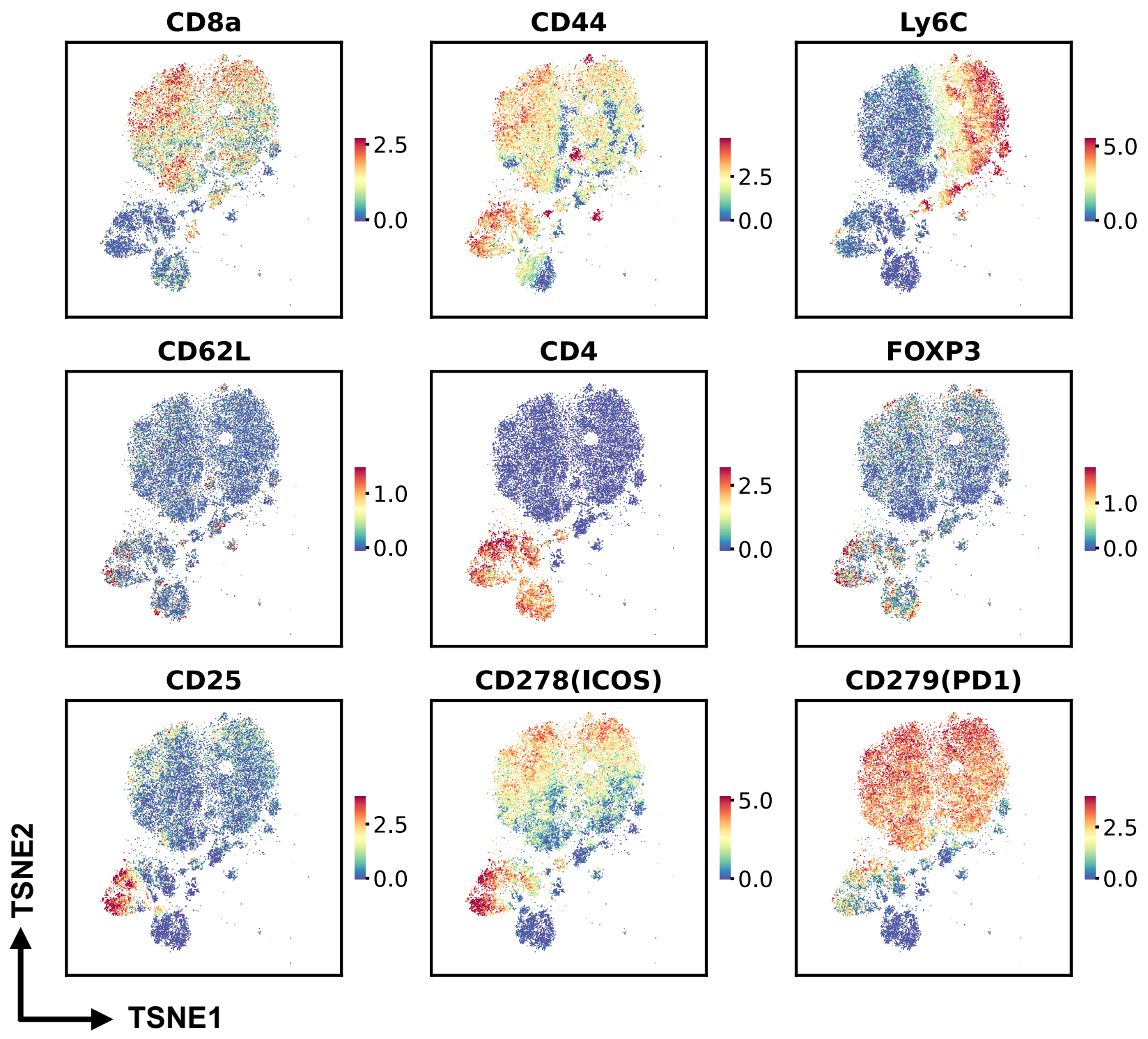

B

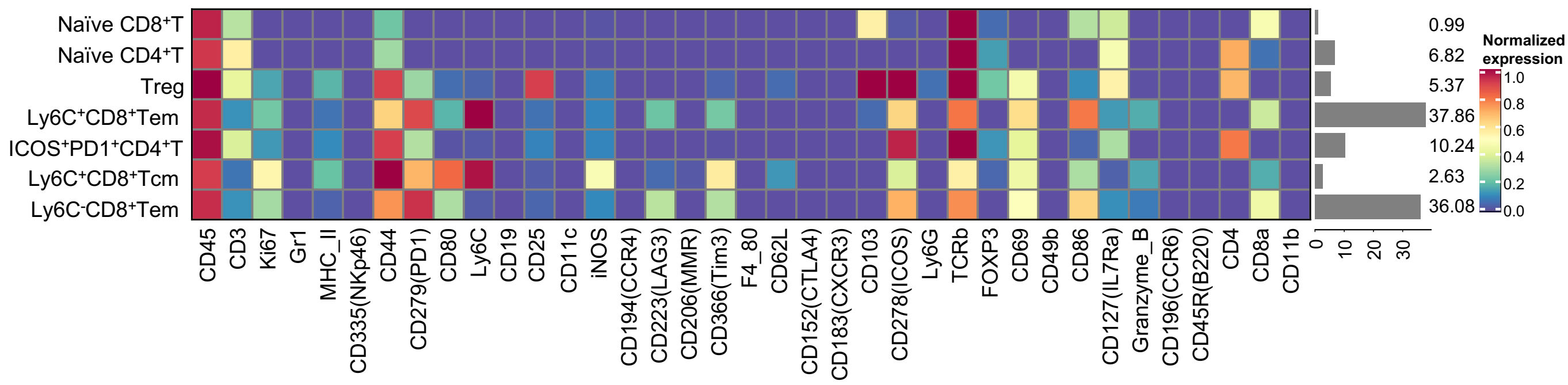

Supplementary Figure 5. IF and flow cytometry analysis of GZMB⁺, perforin⁺, and TNFα⁺ CD8⁺ T cells in mouse tumors following 5-FU/Oxa treatment, with or without autophagy inhibition

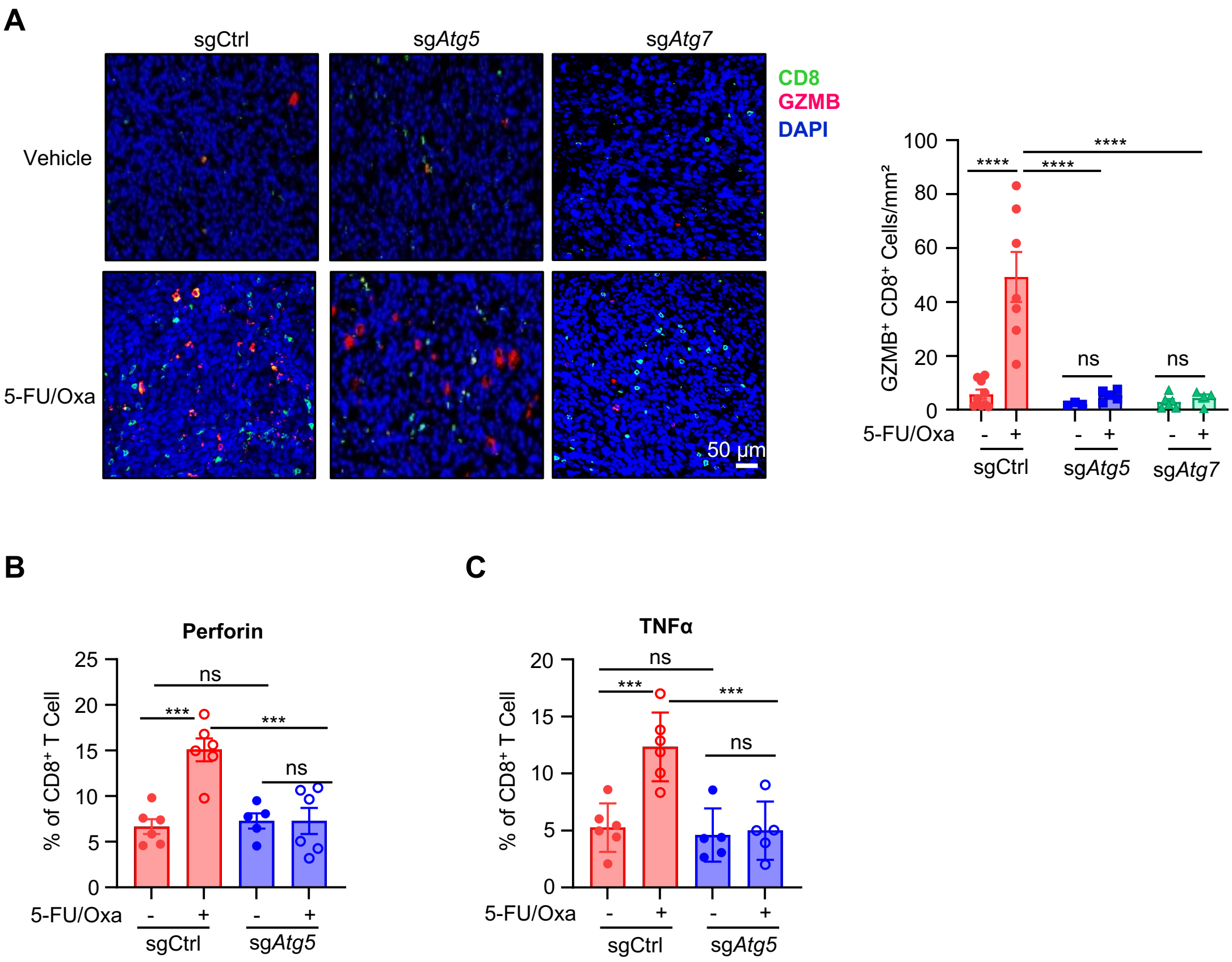

Supplementary Figure 6. Representative images of CFSE and CD69 staining in $CD8^+$ T cells

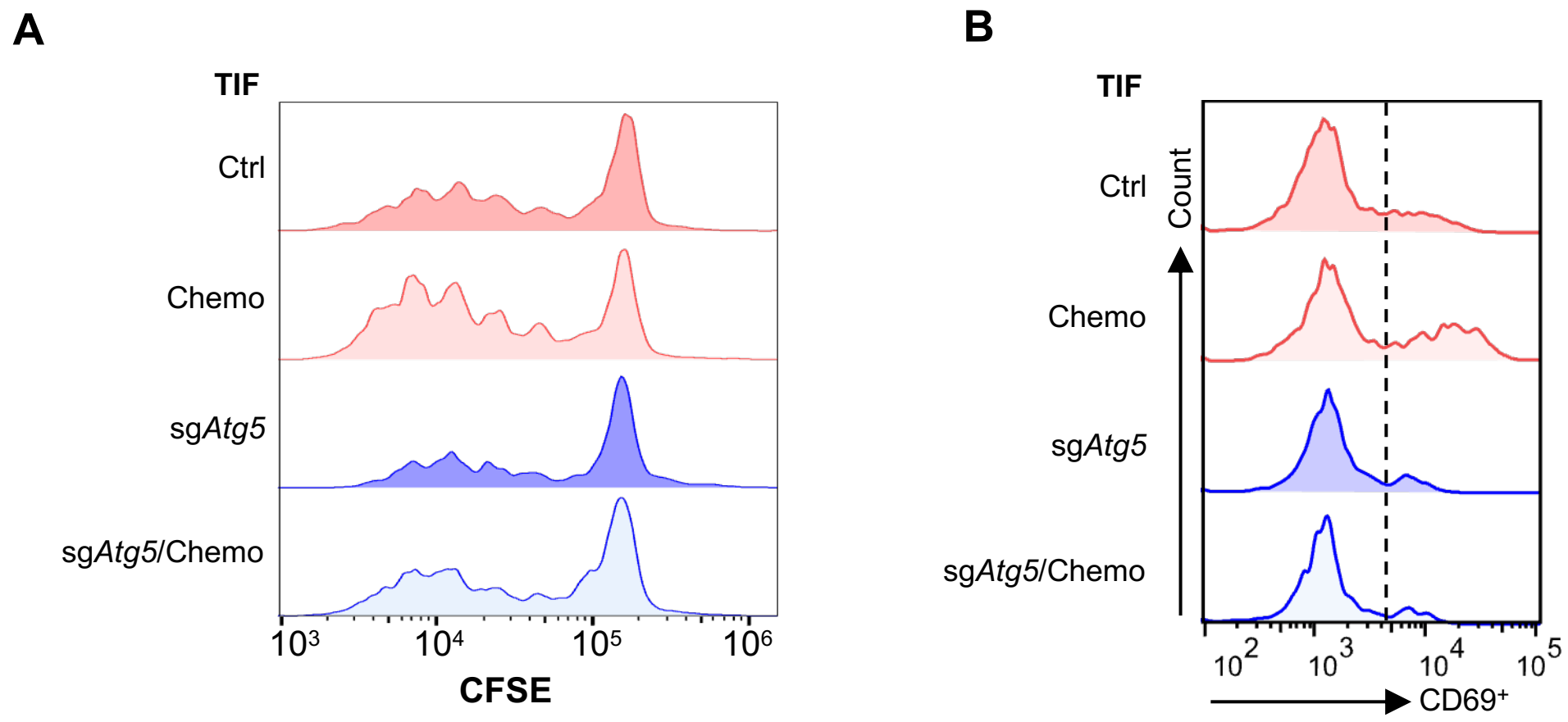

Supplementary Figure 7. RNA-seq analysis reveals downregulation of cytokine signaling pathways upon autophagy inhibition in 5-FU/Oxa-treated cells

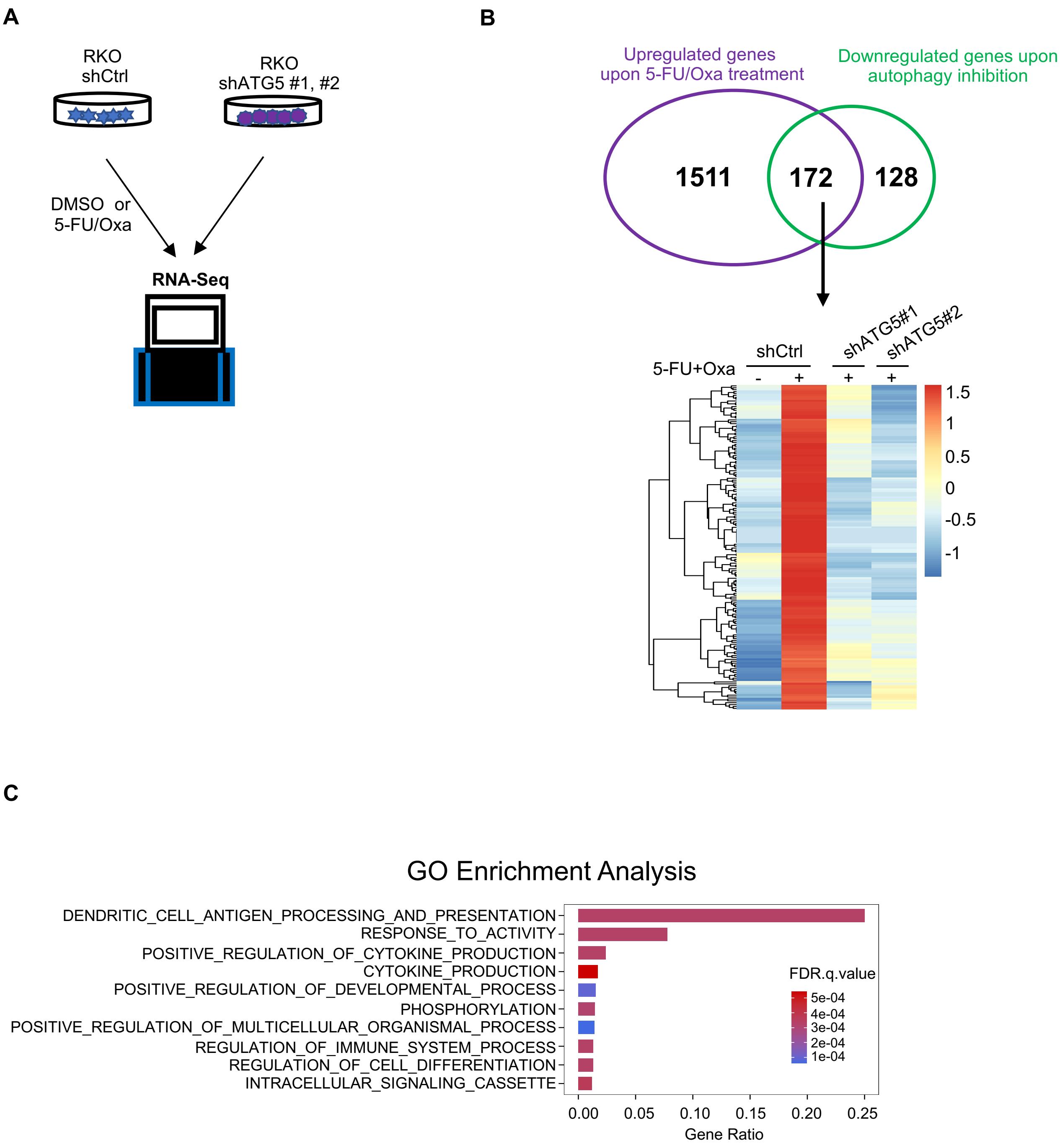

Supplementary Figure 8. TIMP-2 restores GZMB⁺ CD8⁺ T-cell frequencies in autophagy-deficient colorectal tumors following chemotherapy

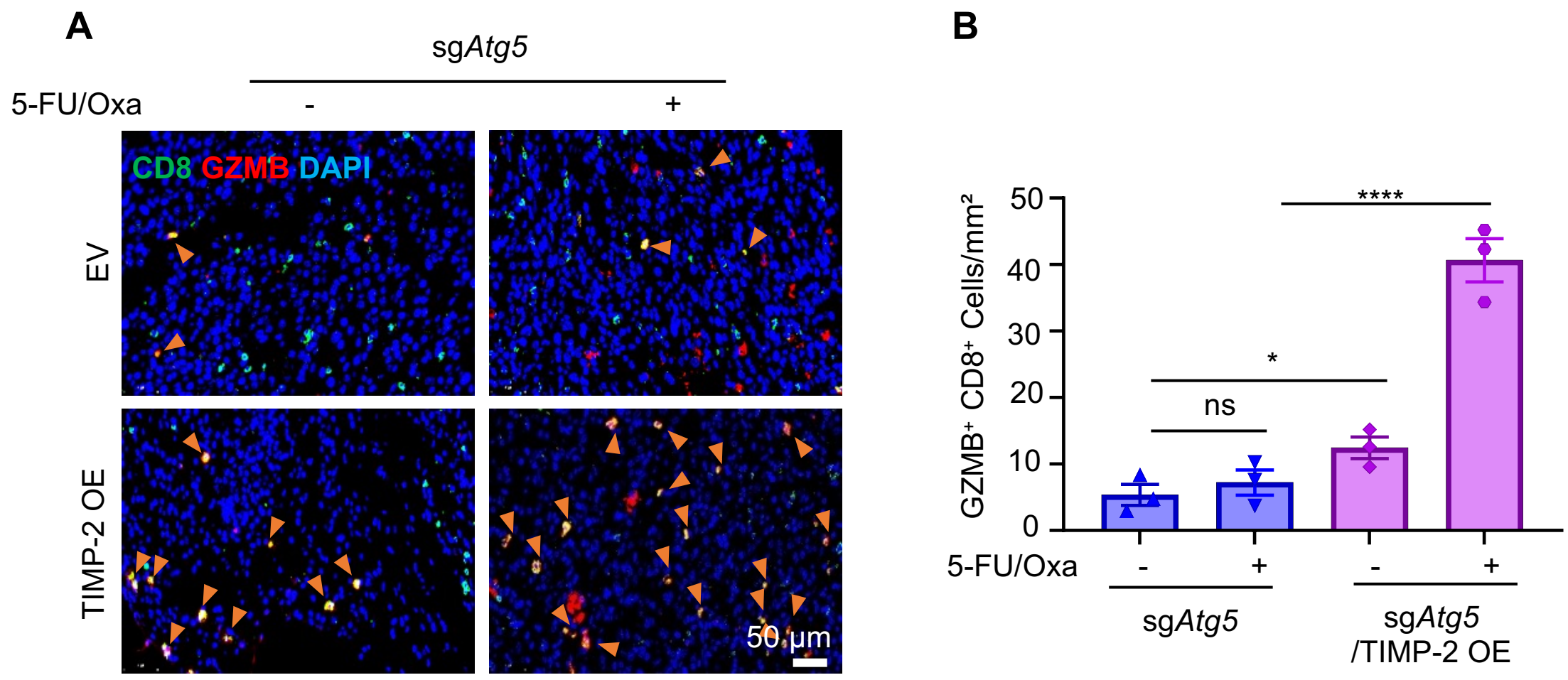

Supplementary Figure 9. The MMP-2/9 inhibitor SB-3CT was well-tolerated in mice

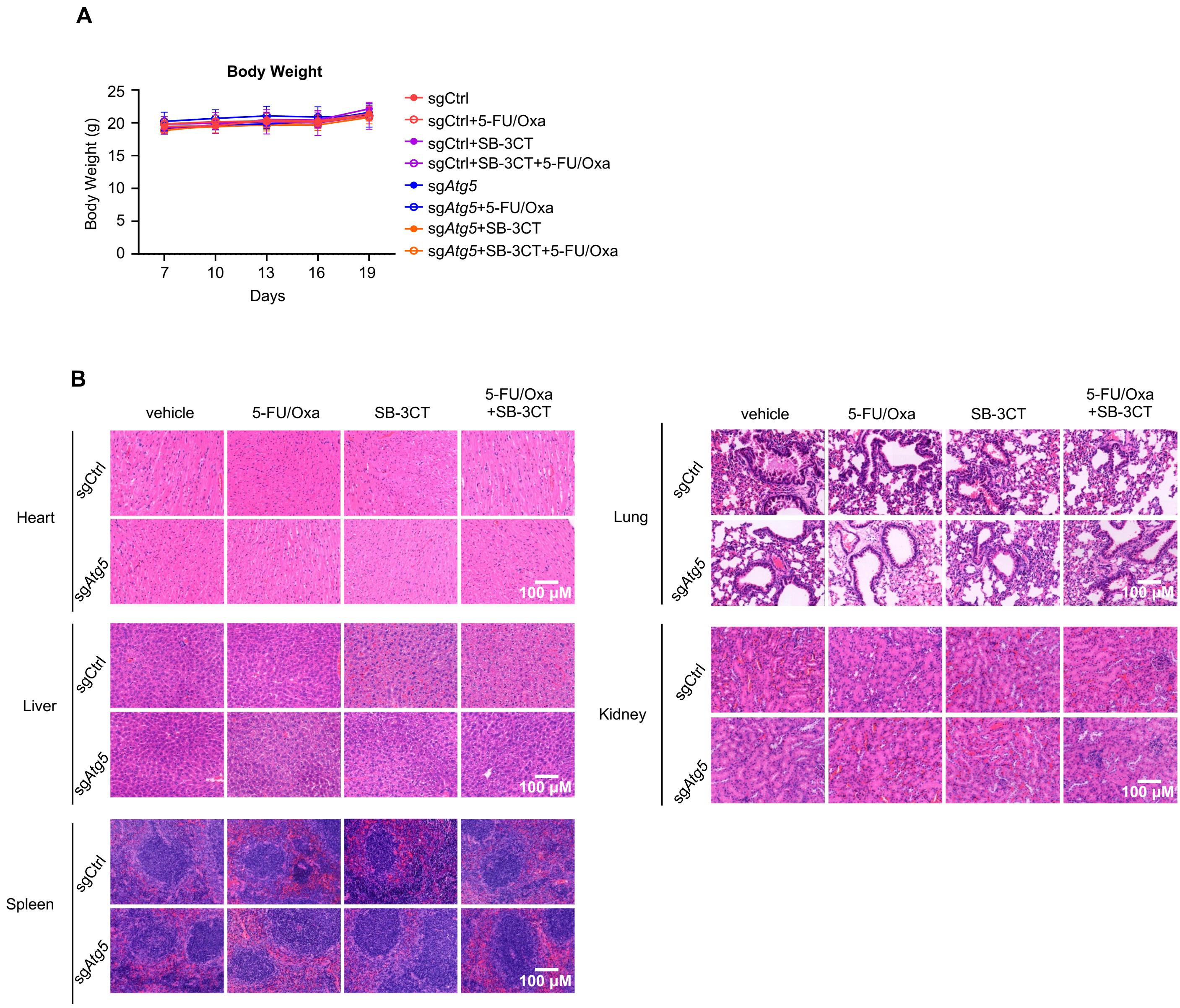

Supplementary Figure 10. In silico prediction of FBXW2–LC3 interaction and experimental validation of their co-localization

A

Protein ID	PSSM Score	Start	End	xLIR
FBXW2	15	54	59	RDFLKL
FBXW2	15	289	294	GDYILL
FBXW2	13	353	358	ASYDIL
FBXL6	9	216	221	LTFLKL
SMURF2	13	726	731	ESYEKL
BTBD1	11	214	219	EGFTDI
IBTK	18	85	90	SGWTAL
IBTK	13	1129	1134	EEFVIV

B

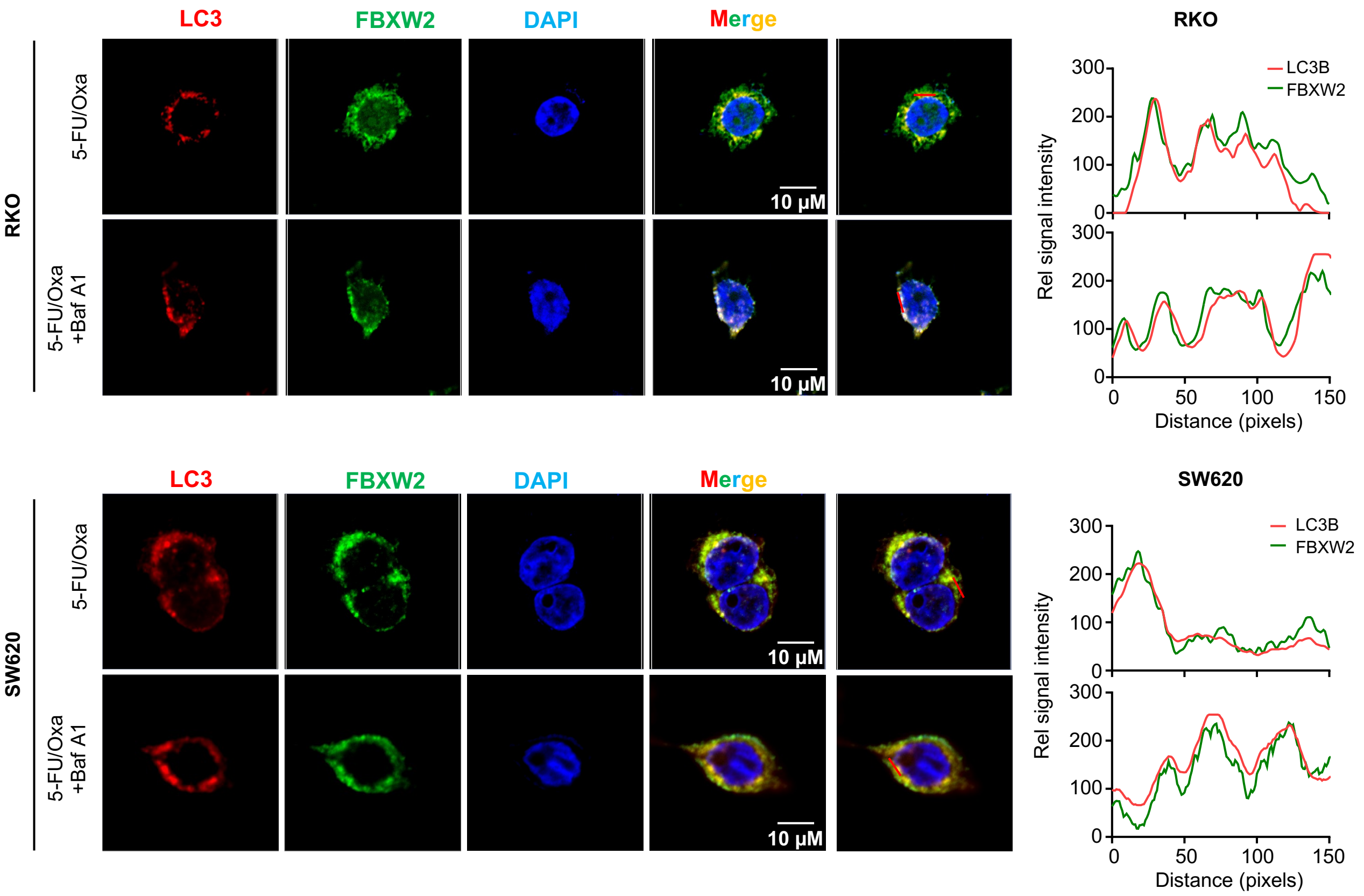

C

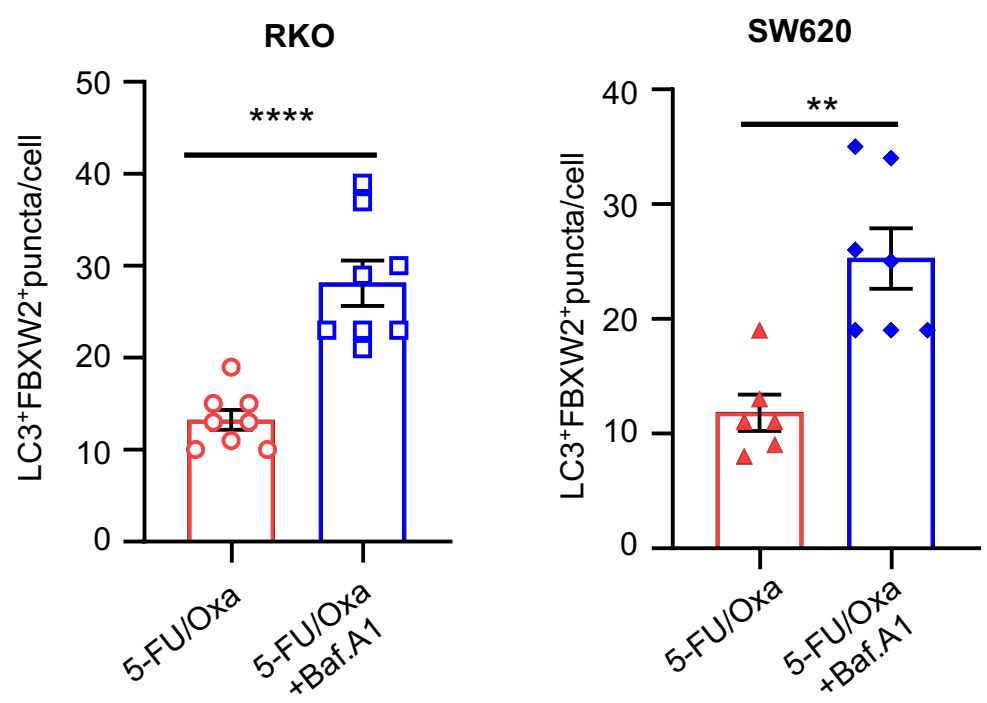

Supplementary Figure 11. Positive correlation between TIMP-2 and anti-tumor immunity

A

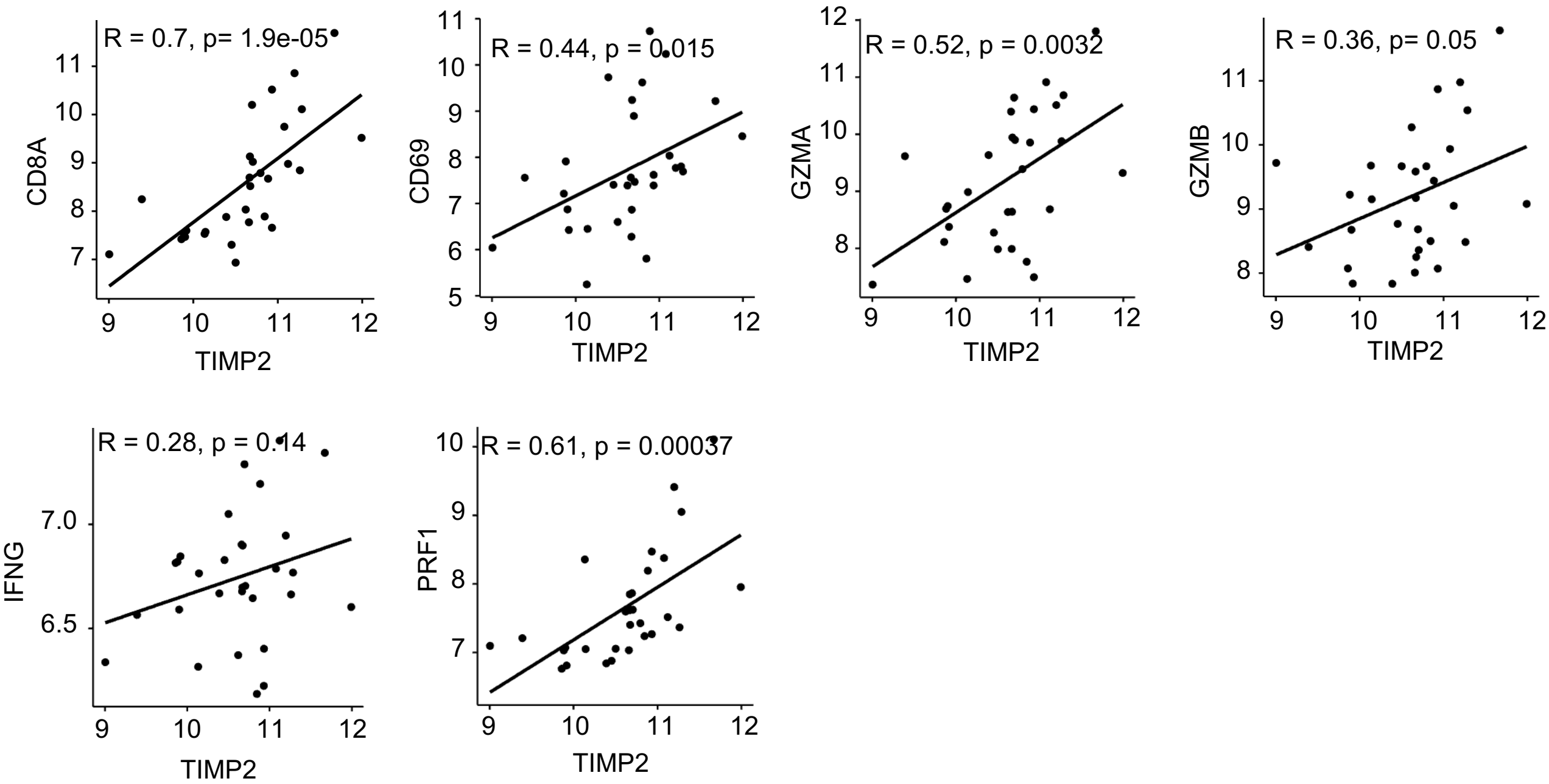

Supplementary Figure 12. Patients with high autophagy or TIMP-2 expression exhibited improved prognostic benefit

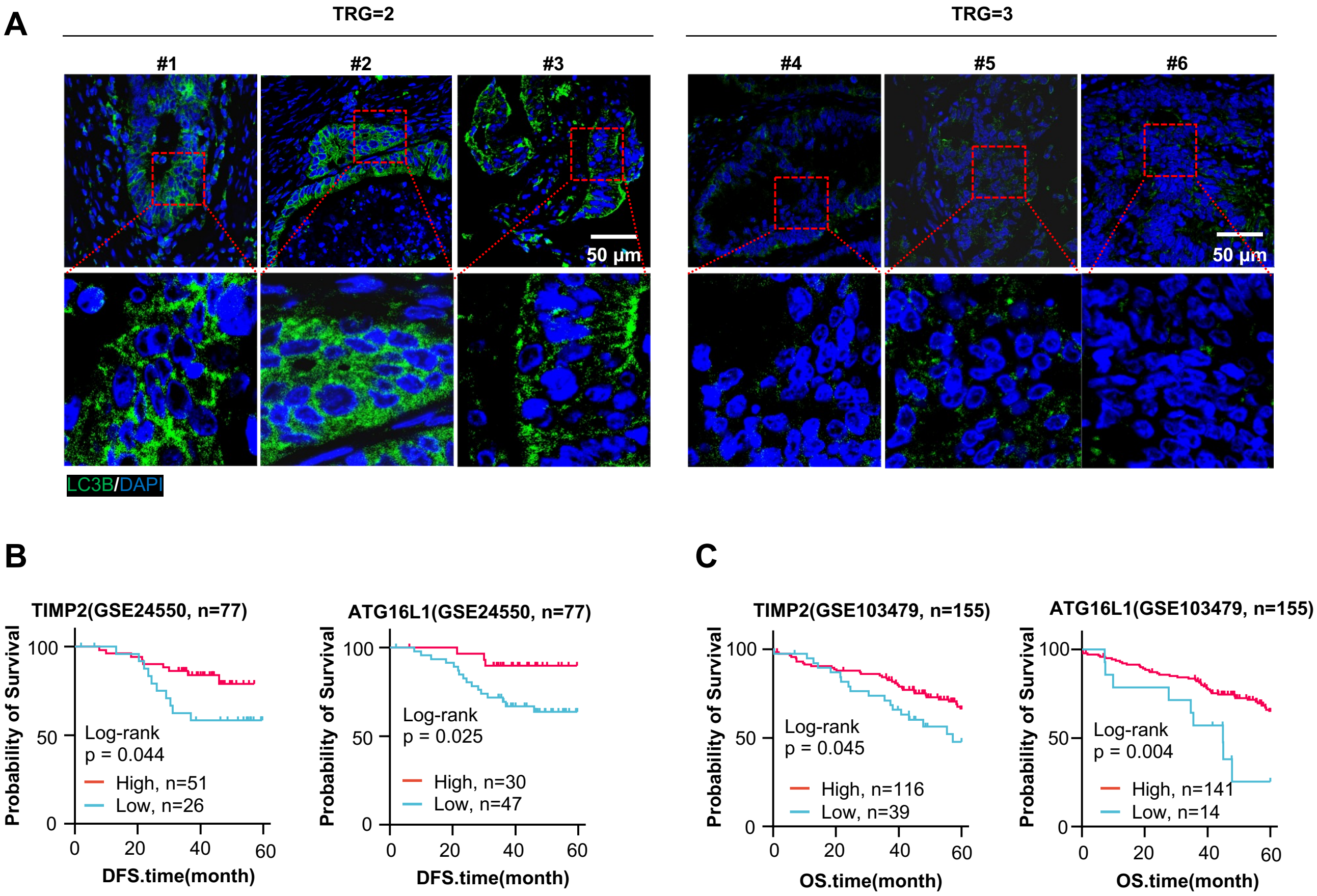

Supplement: Supplementary file 1 [file ADVS-13-e77349-s002.pdf]
