# Supplementary material for: Autophagy Orchestrates Anti‐Tumor Immunity to Enhance Chemosensitivity via the FBXW2/C/EBPβ/TIMP‐2 Axis in Colorectal Cancer
Source: Adv Sci (Weinh). 2026 Aug 21:e77349. Online ahead of print. doi: 10.1002/advs.77349 (PMC13496286; doi:10.1002/advs.77349)
Supplement: Supplementary file 2 [file ADVS-9999-e77349-s001.docx]

**Supplementary methods and materials**

**RNA sequencing and data analysis**

RKO cells with or without autophagy inhibition were treated with 5-FU/Oxa for 24 h. Total RNA was extracted using the RNeasy Mini kit and transcribed to cDNA with random primers. The cDNA was ligated to Illumina sequencing adaptors and sequenced on the Illumina NovaSeq6000 by Novogene(Guangzhou, China). Gene expression was defined by FPKM value (fragments per kilobase of exon per million fragments mapped) using RSEM software. A log2 (fold change) >1 or log2 (fold change) <-0.5 and a false discovery rate (FDR) < 0.01 were used as cut-off thresholds.

Gene Ontology (GO) enrichment analysis was performed using MsigDB. TCGA-COAD dataset was obtained from UCSC Xena (https://xenabrowser.net); GSE69657, GSE45404, and GSE12246 were retrieved from the Gene Expression Omnibus. The autophagy score was calculated as average expression of genes in the gene set GOBP_POSITIVE_REGULATION_OF_AUTOPHAGY (GO:0010508) for each sample.

**Protein mass spectrometry and data analysis**

RKO cells with or without autophagy inhibition were treated with 5-FU/Oxa for 48 h. Cells were washed twice with cold PBS, scraped, and centrifugated at 4 °C for 5 min to obtain cell pellets. Proteins were extracted using SDT buffer (4% SDS, 100 mM Tris-HCl, pH 7.6), followed by digestion with trypsin. 100 μg peptides of each sample were labeled with TMT reagents according to the manufacturer's instructions (Thermo Scientific). LC-MS/MS analysis was conducted on a Q Exactive mass spectrometer (Thermo Scientific). Protein identification and quantification were performed by processing the raw MS data with Proteome Discoverer 2.4, employing the integrated MASCOT search engine (v2.2, Matrix Science) against the UniProt human protein database. Protein ratios were derived from the median intensity of their corresponding unique peptides. Differentially expressed proteins were defined using the criteria of p-value < 0.05 and an absolute log₂ fold change |log₂FC| > 0.25.

**Clinical specimens and immunohistochemistry (IHC)/** **Multiplex immunofluorescence staining**

Clinical specimens were obtained from the Tissue Bank with approval from the Human Medical Ethics Committee of Sun Yat-Sen University and with written informed consent from all patients for the use of their data. Paraffin-embedded specimens of 48 CRC patients who have undergone neoadjuvant chemotherapy with tumor regression grade (TRG) pathologically confirmed were included. In brief, slides were deparaffinized, rehydrated and antigens were retrieved by EDTA solution (PH = 9.0) (Service bio, China) using microwave. The co-staining of Pan-CK, p-ATG16L1(Ser278), LC3B, TIMP-2, and GZMB was performed using Multiplex Immunohistochemistry Kit according to the manufacturer’s instruction (#abs50015, Absin). We evaluated the correlations of TIMP-2 protein expression with the proportion of GZMB-positive cells, as well as the average numbers of p-ATG16L1 and LC3B puncta per cell, in each sample. Based on the median expression levels of p-ATG16L1, LC3B, and TIMP-2, patients were stratified into high- and low-expression groups for each marker. Subsequently, we examined the associations between clinicopathological features and the combined staining patterns of p-ATG16L1/TIMP-2 or LC3B/TIMP-2.

For IHC quantification, staining intensity was analyzed via color deconvolution to separate the RGB channels of the DAB signal. The average integrated optical density (AOD) was determined using the formula: (OD = log(maximum intensity) / measured intensity), where maximum intensity was defined by background tissue-free regions. For Fluorescence Intensity Quantification, individual fluorescence channels were extracted from the raw image files using ImageJ. For each channel, background fluorescence was subtracted by measuring a cell-free region. The mean fluorescence intensity (MFI) of the target region of interest (ROI) was then calculated and normalized against the background-corrected values. To quantify intracellular puncta, images were randomized and blinded to eliminate observer bias. The number of puncta per cell was manually counted across at least 30 randomly selected cells per group using the ImageJ.

**TIF collection and CD8^+^ T activation**

Tumor interstitial fluid (TIF) was collected as described previously[1], briefly 0.3 g tumor was minced and incubated with 200 µL PBS for 10 min at 4°C, then tumor was centrifuged at 4°C and 106 g for 10 min. The TIF was collected and stored at -80°C. For the OT-I T cell activation assay, 5 × 10⁵ OT-I cells were seeded per well in a 24-well plate with 500 uL culture medium. The cells were pretreated with 10 µL of either native TIF, TIF pre-incubated with an anti-TIMP2 antibody (αTIMP2), or TIF pre-incubated with recombinant TIMP2 (rTIMP2) for 30 min, respectively. Following the pretreatment, all groups were stimulated with 1 µg/mL OVA-G4 peptide. After an additional 24 h of culture, CD8⁺ T cell activation was assessed by flow cytometry, gating on CD45⁺ CD8⁺ cells and analyzing CD69 expression.

**T Cell Intracellular Cytokine Staining**

To evaluate T cell effector function, OT-I T cells were activated under the designated conditions. Prior to harvest, cells were treated with Brefeldin A (BFA, 5 µg/mL) for 4 h to block cytokine secretion. Cells were then collected and stained with fluorochrome-conjugated antibodies against cell surface markers (CD45, CD8). After surface staining, cells were fixed, permeabilized using a commercial fixation/permeabilization kit, and stained intracellularly for Granzyme B (GZMB), Perforin, IFN-γ and TNF-α. Cytokine production was quantified via flow cytometry.

**In Vitro Tumor Cell Killing Assay**

The cytotoxic activity of conditioned OT-I T cells against tumor cells was evaluated using a luciferase-based killing assay. OT-I T cells were thawed and seeded at a density of 6 × 10⁵ cells per well in a 48-well plate. Cells were cultured for 48 h in complete RPMI-1640 medium (supplemented with 10% FBS, 1× β-mercaptoethanol, and 10 U/mL IL-2) in the presence or absence of 1 µg/mL OVA peptide and 4 µL of TIF sample per well (n = 3 per group).

Concurrently, MC38-LUC-OVA target tumor cells were seeded into a white 96-well plate at a density of 5,000 cells per well and allowed to adhere overnight. The pre-activated OT-I T cells were then harvested, counted, and added to the MC38-LUC-OVA cells at effector-to-target (E: T) ratios of 2:1. After 24 h of co-culture, bioluminescence imaging was performed to measure luciferase activity, and the percentage of specific tumor lysis was calculated relative to the control wells.

**Supplementary References:**

1.	Reinfeld, B.I., et al., *Cell-programmed nutrient partitioning in the tumour microenvironment.* Nature, 2021. **593**(7858): p. 282-288.

**Supplementary Tables**

**Table S1. Clinicopathologic features of study patients.**

**Table S2**. **Reagents used in this study.**

| **Gene** | **Catalog number** | **Company** |
| --- | --- | --- |
| Recombinant TIMP-2 | CC68 | Novoprotein |
| Human TIMP-2 ELISA Kit | EK0522 | Boster |
| Mouse TIMP-2 ELISA Kit | EK0955 | Boster |
| Mouse TIMP-2 DuoSet ELISA KIT) | DY6304-05 | R&D |
| DuoSet ELISA Ancillary Reagent Kit 2 | DY008B | R&D |
| MG132 | M7449 | Sigma |
| CHX | HY-12320 | MCE |
| Bafilomycin A1 | B1793 | Sigma |
| Brefeldin A | S7046-5mg | Selleck |
| OVA-G4-peptide | RP20398 | GeneScript |
| SB-3CT | T2009 | TargetMol |
| 5-FU | F6627 | Sigma |
| Oxaliplatin | 9512 | Sigma |
| Chloroquine | 6628 | Sigma |
| Recombinant MMP-9 | 11602-MP-020 | Novus |
| Corn oil | HY-Y1888 | MCE |
| Dimethyl sulfoxide | HY-Y0320 | MCE |

**Table S3**. **The sequences of sgRNA, shRNA, siRNA**

| **Name** | **Primer Sequence 5'--> 3'** |
| --- | --- |
| Mouse *Atg5* sgRNA#1 | AGTGAAAAAGCACTTTCAGA |
| Mouse *Atg5* sgRNA#2 | AACGTCAAATAGCTGACTCT |
| Mouse *Atg7* sgRNA#1 | ACGTCCAGGGCACTATTAAA |
| Mouse *Atg7* sgRNA#2 | GAAACTTGTTGAGGAGCAT |
| Mouse *Timp*-2 sgRNA#1 | AGCAGCTGCAGGCGTCGGCC |
| Mouse *Timp*-2 sgRNA#2 | CGCCTGTTGCGGGTGCACCG |
| Mouse *Cebpb* siRNA#1 | ACAAGGUGCUGGAGCUGAC |
| Mouse *Cebpb* siRNA#2 | CACCGGGUUUCGGGACUUG |
| Mouse Control sgRNA#1 | GAGGCGGGCTCTTCTATCGT |
| Mouse Control sgRNA#2 | CATACCCGGTGTTTCCTAGC |
| Mouse Control siRNA | UUCUCCGAACGUGUCACGU |
| Human *ATG5* shRNA#1 | CCTTTCATTCAGAAGCTGTTT |
| Human *ATG5* shRNA#2 | CCTGAACAGAATCATCCTTAAG |
| Human Control shRNA | GCGAGGTATTCGGCTCCGCG |
| Human *CEBPB* siRNA#1 | UGCCUUUAAAUCCAUGGAA |
| Human *CEBPB* siRNA#2 | ACUUCCUCUCCGACCUCUU |
| Human *FBXW2* siRNA#1 | GCCUUUGAAACCUCGUCAUUA |
| Human *FBXW2* siRNA#2 | GCAGCGGUGAAGUUUGAUGAA |
| Human Control siRNA | ACGUGACACGUUCGGAGAA |

**Table S4. The primers for qRT-PCR, CHIP-PCR and plasmids construction**

| **Gene** | **Forward Primer 5'--> 3'** | **Reverse Primer 5'--> 3'** |
| --- | --- | --- |
| Mouse *Timp*-2 | AGCCAAAGCAGTGAGCGAGAAG | GCCGTGTAGATAAACTCGATGTC |
| Mouse β-actin | CATTGCTGACAGGATGCAGAAGG | TGCTGGAAGGTGGACAGTGAGG |
| Human TIMP-2 | ACCCTCTGTGACTTCATCGTGC | GGAGATGTAGCACGGGATCATG |
| Human CEBPB | AGAAGACCGTGGACAAGCACAG | CTCCAGGACCTTGTGCTGCGT |
| Human FBXW2 | GCCAGAGTGTATGCACTTTACT | TGTTCATCAAACTTCACCGCT |
| Human β-actin | CACCATTGGCAATGAGCGGTTC | AGGTCTTTGCGGATGTCCACGT |
| CHIP-PCR-BS1 | AGCACCGCACAATTTAGTGG | ACACAAGAAATTAGCCGGGC |
| CHIP-PCR-BS2 | TGGCCAGGCGCACTTAAAAT | CCAAGGGGTGTCCTTTGGAG |
| TIMP-2 promoter FL | CGACGCGTCAGCATTTTGTTCCCCCTG | CCCTCGAGGCGGGCTTGGGCCGGGCCGCAGT |
| TIMP-2 promoter BS1 Del | ATACACAAACAGAAAGAAGCT | CTTTTCTCAGAACTGTCT |
| TIMP-2 promoter BS2 Del ion-TIMP-BS2 | AGGCCACCCGCCCACAGAAAC | CCATTTGAAAAAGGGATCCTGTC |
| *Timp*-2 Ala + mt | GAAGCTGCAGCTGCTCCCCGGTGC | GGCGTCGGCCGGGCGCAGCAGCGTG |
| *Timp*-2-C | GAGTGCAAGATCACTCGCTGTC | GGCGTCGGCCGGGCGCAGCAGCGTG |

**Table S5**. **Antibodies used in the study**

| **Name** | **Catalog number** | **Company** |
| --- | --- | --- |
| ATG5 | 12994 | CST |
| ATG7 | 10088-2-AP | Proteintech |
| TIMP-2 | 5738/17353-1-AP | CST/Proteintech |
| LC3B | 7543/14600-1-AP | Sigma/Proteintech |
| SQSTM1/P62 | ab91526/sc-28359  sc-28359 | Abcam/Santa Cruz |
| CEBPB | 23431-1-AP/ sc-7962 | Proteintech/Santa Cruz |
| FBXW2 | 11499-1-AP | Proteintech |
| β-actin | A5441 | Sigma |
| HRP-conjugated secondary goat anti-rabbit | A6154 | Sigma |
| HRP-conjugated secondary donkey anti-mouse | A4416 | Sigma |
| [Mouse Anti-Rabbit IgG LCS](https://www.abbkine.com/product/ipkine-hrp-mouse-anti-rabbit-igg-lcs-a25022) | A25022 | akkbine |
| [Goat Anti-mouse IgG LCS](https://www.abbkine.com/product/ipkine-hrp-mouse-anti-rabbit-igg-lcs-a25022) | A25012 | akkbine |
| CD8 | 853360/ 563234 | CST/BD |
| CD45 | 70257/103116 | CST/Biolegend |
| p-ATG16L(S278) | ab195242 | Abcam |
| Granzyme B | 17215/372220 | CST/ Biolegend |
| BV650 Rat Anti-Mouse CD8a/FITC Rat Anti-Mouse CD8a | 563234 | BD |
| APC/Cyanine7 anti-mouse CD45 Antibody | 103116 | Biolegend |
| PE-Cy™7 Hamster Anti-Mouse CD3e | 561100 | BD |
| eBioscience Fixable Viability Dye eFulor 520 | 65086714 | Invitrogen |
| PE/Dazzle™ 594 anti-mouse IFN-γ | 505845 | Biolegend |
| Alexa Fluor® 647 anti-human/mouse Granzyme B | 372220 | Biolegend |
| Brilliant Violet 605™ anti-mouse Ly-6C | 128035 | BD |
| Brilliant Violet 421™ anti-mouse CD335 (NKp46) | 137611 | Biolegend |
| PE Hamster Anti-Mouse CD69 | 553237 | BD |
| APC anti-mouse Perforin Antibody | 154303 | Biolegend |
| Brilliant Violet 421™ anti-mouse TNF-α | 506328 | Biolegend |
| Pan-CK | 4545 | CST |
| Alexa 488 goat-anti-rabbit IgG H+L | 11008 | Thermo Fisher |
| Alexa 594 goat-anti-rabbit IgG H+L | 11012 | Thermo Fisher |
| Alexa 488 goat-anti-mouse IgG H+L | 11001 | Thermo Fisher |
| Alexa 594 goat-anti-mouse IgG H+L | 11005 | Thermo Fisher |
| CD3 | 317308 | Biolegend |
|  |  |  |
|  |  |  |
| Flag | F1804 | Sigma |
| HA | 3724/GTX115044 | CST/Genetex |
| His | ab18184 | Abcam |
| Mouse Anti-Rabbit IgG LCS | A25022 | Abbkine |
| Goat Anti-Mouse IgG LCS | A25012 | Abbkine |
